# Identification and Characterization of a Glucometabolic Prognostic Gene Signature in Neuroblastoma based on N6-methyladenosine Eraser ALKBH5

**DOI:** 10.7150/jca.69408

**Published:** 2022-03-28

**Authors:** Kezhe Tan, Wei Wu, Kai Zhu, Li Lu, Zhibao Lv

**Affiliations:** Department of General Surgery, Shanghai Children's Hospital, Shanghai Jiao Tong University, Shanghai, China.

**Keywords:** neuroblastoma, m6A eraser, ALKBH5, glucometabolic genes, prognostic model, drug targets.

## Abstract

**Background:** Neuroblastoma (NB) is a pediatric cancer occurring in the peripheral nervous system. A demethylase, alkylation repair homolog protein 5 (ALKBH5), is one type of N6-methyladenosine (m6A) eraser that plays a tumor-suppressive role in a variety of cancers. The significance of carbohydrate metabolism in cancer has been intensively investigated over the years, but the correlation between ALKBH5 and glucose metabolism in NB remains to be elucidated.

**Methods:** Based on the overlapped genes (DE-GRGs) of ALKBH5-related differentially expressed genes (ALKBH5-DEGs) in GSE62564 (n=498) and genes related to glucose metabolism (GRGs), a LASSO regression model was constructed. External validations with datasets (EGAS00001001308, n=139 & GSE16476, n=88) and the NB samples from Shanghai Children's Hospital (SCH) were performed. Meanwhile, biological and clinical utility, immune cell subtypes and drug sensitivity were assessed.

**Results:** ALKBH5 was significantly correlated with better overall survival (OS) in NB patients, and gene set enrichment analysis (GSEA) showed its enrichment in GO/ KEGG terms regarding glucose metabolism. 27 of the 31 DE-GRGs were included in the LASSO screen after the univariate analysis. A prognostic glucometabolic model including AHCY, NCAN, FBP2, GALNT3 and AKR1C2 was established with the internal and external validation with biological experiments: the high-risk subtype compared to the low-risk subtype showed oncogenic and MYCN-related malignancy, glucometabolic dysregulation, poor prognosis and immunosuppression. TGX-221 was predicted to be a potential therapeutic drug and validated to suppress NB oncogenes including MYCN, AHCY and NCAN and immunosuppressive DNMT1 in NB cells.

**Conclusion:** ALKBH5 was closely related to glucometabolic processes, and our prognostic model had high application value in predicting & assessing the OS of NB patients, and even served potential drug targets.

## Introduction

Neuroblastoma (NB), the most common extracranial solid childhood tumor in infants and children, accounts for 8~10% of childhood cancers and 15% of deaths in children 1. Based on age, the International Staging System (INSS), histology, DNA copy number, MYCN amplification and other characteristics, NB patients are classified into very low-, low-, intermediate- and high-risk groups according to Children's Oncology Groups (COGs) 2, 3. According to statistics across various countries, the overall survival (OS) rate of high-risk NB is still below 50% 1; therefore, the final goal of NB research is to improve the quality of life and survival of high-risk NB patients. Abnormalities in genes such as MYCN, ALK, TERT, PHXO2B, and ATRX have come to be regarded as high-risk factor for NB; however, high-risk NB is characterized by heterogeneous genetics, and more than 25% of high-risk NB patients do not show the abovementioned genetic abnormalities 4, 5. Therefore, more biomarkers need to be identified to improve the current clinical evaluation system.

The N6-methyladenosine (m6A) modification plays a role in cancer-related biological processes, including proliferation, invasion and metastasis 6, 7. The regulation of this m6A involves readers such as IGF2BP3 and YTHDF2, writers such as METTL3 and METTL14 and erasers such as ALKBH5 and FTO 8. There are a few reports concerning m6A writers 9, 10 and readers 11, 12 in NB, whereas the role of m6A erasers in NB remains to be elucidated. We briefly investigated the m6A erasers ALKBH5 and FTO in a dataset including survival data and found that ALKBH5 might play a more prominent role in tumor suppression. Therefore, our study aimed to interpret the biological role of ALKBH5 in NB.

Our preliminary gene set enrichment analysis (GSEA) of the ALKBH5-low and ALKBH5-high groups in the dataset suggested that glucometabolic processes might play an essential role. Cancer development is characterized by aberrant glucometabolic processes, as aerobic glycolysis (the Warburg effect) has been intensively studied over past decades 13, 14. Nevertheless, there are no available reports concerning ALKBH5 and carbohydrate metabolism in NB.

Therefore, we performed integrative bioinformatic analyses in the present study to identify ALKBH5 and correlate its biological mechanism with carbohydrate metabolism. Based on a combination of univariate Cox regression and least absolute shrinkage and selection operator (LASSO) regression, a prognostic model including 5 glucometabolic genes (AHCY, GALNT3, AKR1C2, NCAN and FBP2) was established and proven to be effective in predicting NB patient prognosis in different cohorts. A brief experimental validation was also performed in NB tissue samples from our own center and MYCN-amplified BE(2)-C cells. Additional biological and clinical values of the glucometabolic gene signature were investigated, and potential drug targets were predicted and tested in genomics of drug sensitivity in cancer (GDSC). By elucidating the intricate relationships among ALKBH5 and these 5 predictive factors, our study provides a new combination of biomarkers for the prognostic management of NB patients and the PI3K-β inhibitor TGX221 might be possibly effective in suppressing NB by finetuning the aberrant glucose metabolic processes.

## Methods and Materials

The retrospective study was approved by the Institutional Review Board (IRB) of Shanghai Children's Hospital (SCH), Shanghai Jiao Tong University, in accordance with the principles of Declaration of Helsinki. Written informed consent to participate in this study was provided by the participants' legal guardian or next of kin. Patient's identities and privacy were protected and invisible in the study.

### Study Design and Public Data Acquisition

There were currently more than 50 NB public datasets available, but we priorly selected RNA-Seq rather than microarray in transcriptome profiling because the benefits of RNA-Seq over microarray, such as superiority in detecting low abundance transcripts and more compatibility in performing immune infiltration analysis 15. For these considerations, the inclusion criteria of training set were that the NB RNA-Seq samples must originate from the human tissues with the sample number over 200 and the complete information of survival state and time. The exclusion criteria were that the NB patients suffered other diseases except for NB. Eventually, we selected NB RNA-seq dataset GSE62564 (n=498) with the largest sample numbers and the most complete clinical and survival information for further analysis. GSE62564 was generally used for analysis of m6A erasers, GSEA, gene signature development, ssGSEA, etc. Also, the GSE62564 were randomly divided into a training set (TS, n=349) and an internal validation set (IVS, n=149) at the ratio of 7:3. The inclusion criteria of external validation datasets were that NB samples must have the gene expression profile concerning the newly constructed glucometabolic model in the training set, and the exclusion criteria were existed non-NB diseases. Therefore, the NB RNA-seq dataset EGAS00001001308 (abbreviated as EGAS) and microarray dataset GSE16476 were used as external validation sets (EVS), which were obtained from the R2 database (https://hgserver1.amc.nl/cgi-bin/r2/main.cgi). Other microarray datasets generated on the GPL570 platform for different types of specimens, including normal adrenal glands (normal AG; GSE3526, GSE7307 and GSE8514) and NB tissue samples (GSE13136, 14880, 16237 and 16476), were also extracted from the R2 database. Cancer Cell Line Encyclopedia (CCLE) data (version 21Q3) were downloaded from https://depmap.org/portal/.

For the acquisition of genes related to glucose metabolism (GRGs), all genes in the h.all.v7.2.entrez.gmt gene set (downloaded from the GSEA official website) were subjected to enrichment analysis. Finally, 384 genes enriched in KEGG pathways/GO terms related to glucose metabolism (FDR q-value < 0.25) were obtained, which were considered to be GRGs. Detailed information about the enrichment analysis is provided in **Supplementary Table 1**.

### Patients and Specimens

Fourteen primary NB samples according to the INSS (n=7 in T1, T2 and T4S **vs.** n=7 in T3 and T4) from SCH were obtained between January 2015 and December 2019. These tissue samples were snap-frozen in liquid nitrogen and then kept at -80°C.

### GSEA

To explore the underlying molecular mechanism of ALKBH5, 498 pediatric NB patients were classified into ALKBH5-high (n=249) and ALKBH5-low (n=249) groups based on the median log_2_RPM value. GSEA software (version_4.1.0) was used to investigate GO terms and the KEGG pathways between these two groups. The GO terms belonged to three major categories: biological process (BP), cellular component (CC), and molecular function (MF). Meanwhile, glucometabolic gene sets enriched in **Supplementary Table 1** were also used for further analysis. In addition to GO and KEGG analysis, Hallmark gene set (50 gene sets, v7.2) and some common MYCN-associated gene sets including NMYC_1, KIM_MYCN_AMPLIFICATION_TARGETS_UP as well as LAST--OWSKA_COAMPLIFIED_WITH_MYCN were applied. A false discovery rate (FDR) < 0.25 was regarded as statistically significant.

### Survival Analysis and Identification of Differentially Expressed Genes (DEGs)

All survival data were analyzed and presented in Kaplan-Meier curves (package “survival” and “survminer” in R). P < 0.05 was deemed to be statistically significant. The hazard ratio (HR) was used to evaluate risk factors, where HR>1 indicated risk, and HR<1 indicated protection. The DEGs between the ALKBH5-high and ALKBH5-low groups were analyzed using a t-test. The cutoff was set for DEG selection based on the criterion of P < 0.05 and absolute value of log_2_ (fold change, FC) > 0.5.

### Venn Diagram and Functional Enrichment of Overlapped ALKBH5-DEGs and GRGs (DE-GRGs)

Venn diagram analysis was conducted using the available online tool (http://bioinformatics.psb.ugent.be/webtools/Venn/). The group of overlapping ALKBH5-DEGs and GRGs was referred to as DE-GRGs. To reveal the functions of DE-GRGs, GO terms and KEGG pathway enrichment analyses were performed using the “clusterProfiler” package in R (**Supplementary Table 2**)**.** The top 5 items were visualized in Figures.

### Gene-gene Correlation, Protein-protein Interaction (PPI) Network and Cytoscape Analyses

To further clarify the interaction among the DE-GRGs, the gene-gene correlation value was analyzed using the Pearson method. The GeneMANIA tool (https://genemania.org/) was used to analyze gene-gene networks and functions by applying all available online databases/libraries. The edge (link) count was downloaded and analyzed by Cytoscape (Version 3.8.2) to determine the hub genes. In addition, the STRING (http://string-db.org/) database was applied to determine the PPI network of DE-GRGs with an interaction score of 0.15, after which Cytoscape combined with the Cytohubba plugin was used to visualize the PPI network and hub genes.

### Prognostic Gene Signature Establishment

A total of 498 patients in GSE62564 with expression and survival data were enrolled in the analysis. First, 31 DE-GRGs were analyzed via univariate Cox regression to screen out prognostic genes (HR>1 or <1, P<0.05) in the whole dataset. Thereafter, these patients were randomly divided into TS and IVS at a ratio of 7:3. We constructed a prognostic model of a multiple gene signature by performing a LASSO regression analysis of prognostic DE-GRGs using the “glmnet” package in R. The DE-GRG signature could be used to predict the risk score as follows:

Risk score = 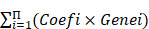


### Clinical Evaluation of Gene Signature

NB Patients were classified into a high-risk and low-risk subtype based on the median LASSO risk scores (LRS) of TS, IVS and EVS. The predictive value of the gene signature and the survival status of the high- and low-risk subtypes within TS, IVS and EVS were analyzed and visualized based on the time-dependent receiver operating characteristic (ROC) curve (package “timeROC” and “ggplot2” in R) and the Kaplan-Meier curve.

In addition to investigating whether the prognostic gene signature could be independent of other clinical parameters (including age, sex, INSS and MYCN level), univariate and multivariate Cox analyses were conducted (package “survival” in R). All independent prognostic factors identified as significant by multivariate Cox regression analysis were included to build a nomogram and calibration plot to investigate the probability of 3-, 5-, and 7-year OS of NB using the package “rms” in R.

### Q-RT-PCR Analyses

Approximately 25 mg NB tissue samples were weighed and homogenized in 500 µL of TRIREAGENT (Thermo Fisher Scientific, Cat. No: TR 118). RNA isolation was performed according to the manufacturer's manual. RNA amounts and integrity were analyzed with a Nanodrop 2000 spectrophotometer. A total of 500 ng of RNA in a volume of 10 µL per reaction was reverse transcribed into cDNA based on a High-Capacity RNA-to-cDNA Kit (Thermo Fisher Scientific, Cat. No: 4387406). A Q-RT-PCR mixture including cDNA and SYBR green buffer (Thermo Fisher Scientific, Cat. No: A25742) was finally transferred to a 384-well PCR plate (Thermo Fisher Scientific, Cat. No: AB1384), and PCR was conducted on a QuantStudio^TM^ 5 Real-Timer PCR system (Thermo Fisher Scientific, Cat. No: A34322). The appropriate threshold was adjusted according to the amplification plot, and the Ct value was extracted. GAPDH was applied as a housekeeping gene, and the relative transcriptional expression level was calculated according to the 2^delta Ct approach. The mRNA level was normalized to 1 based on the median value in the T1+T2+T4S or MYCN-nonamplification group. The Q-RT-PCR primers are listed in **Supplementary Table 3**.

### Tissue Section and IHC Staining

The NB tissues were fixed with 10% formaldehyde to construct paraffin embedded tissues that were sectioned by professional pathologists in SCH. Immunohistochemical (IHC) staining were performed by Servicebio biotechnology company in Shanghai using the primary rabbit antibody against human ALKBH5 (Proteintech, #16837-1-AP).

### Cell Culture

Neuroblastoma MYCN-amplified cell lines BE(2)-C and IMR-32 were purchased from Chinese Academy of Sciences Cell Bank. BE(2)-C cells were cultured in DMEM/F12 (Gibco, #11330-032) supplemented with 10% fetal bovine serum (FBS; Sigma, #F2442) and 1×Penicillin Streptomycin (P/S) Solution (BasalMedia, #S110JV). IMR-32 cells were cultured in Eagle's Minimum Essential Medium (MEM) (BasalMedia, #L510KJ) supplemented with 10% FBS and 1×P/S. Virus packaging HEK293T cells were cultured in Dulbecco's modified Eagle medium/High glucose (DMEM) (BasalMedia, #L110KJ) added with 10% FBS and 1×P/S. Short Tandem Repeat (STR) sequencing was performed in NB cell lines by BIOWING Biotech Co., Ltd in Shanghai.

### Virus Packaging and Construct of Stable Transfected Cells (STCs)

Lentiviral shRNA was constructed by molecularly cloning target oligonucleotides into the Tetracycline-on (Tet-on) puromycin-resistant plasmid (Addgene, #21915). The DNA extraction of plasmids was performed using DNA extraction kit (Vazyme, DC112-01). The lentivirus was packaged by transfecting objective plasmids with packaging vectors (psPAX2 and pMD2.G) and PEI MAX solution (Polysciences, #24765) in HEK293T cells. The ratio of DNA mass to PEI MAX (1mg/ml stock solution) is 1:3. Afterwards, the virus supernatant was collected, filtered with 0.45μm strainer, concentrated with PEG6000 (Sigma, #81253), resolved in PBS and then aliquoted for subsequent transfection.

Cells were transfected at an approximately multiplicity of infection (MOI) 1.5 to 3 after 48 hours, and the positive STCs were selected after 72 hours of 2μg/mL puromycin (YEASEN, 60210ES25) treatment. 1μg/mL of doxycycline (Dox) was used to induce ALKBH5 knockdown in Tet-on STCs. The target oligonucleotides are listed in **Supplemental Table 3**.

### Immunoblotting

Protein samples were lysed in RIPA buffer (Thermo Fisher Scientific, #89900) and quantified using the Pierce BCA kit (Thermo Fisher Scientific, #23225). The 10-20μg/lane denatured protein samples were segregated by sodium dodecyl sulfate polyacrylamide gel (SDS-PAGE) and transferred onto polyvinylidene difluoride (PVDF) membranes. Afterwards, the 5% fat-free milk (BD Biosciences, #232100) in Tris buffer saline with Tween 20 (TBST) was used to block the membranes and then the primary antibodies rabbit-anti-human ALKBH5 (1:1000; Proteintech, #16837-1-AP) and rabbit-anti-human β-Tubulin (1:5000; Abcam, #ab6046). Secondary antibodies were HRP-conjugate goat anti-rabbit IgG (0.2 ug/ml; Pierce, #31460). The luminescent image analyzer (Fujifilm, LAS-4000) was used to visualize the chemical fluorescence images of proteins after incubation with enhanced chemiluminescence reagents (Tanon, #180-5001).

### Cell viability measured by Cell-Titer-Glo (CTG)

Cells were plated in 96-well plate in triplicate at 1,000 cells/well within 100μL culture medium, and then the CellTiter-Glo® luminescent cell viability assay (Promega, #G7573) was used to assess the cell viability at day 0 and 3 according to the manufacturer's protocol.

### Assessment of Immune Cell Subpopulations

The immune cell abundance identifier (http://bioinfo.life.hust.edu.cn/ImmuCellAI#!/) tool was applied to estimate immune cell subpopulations using the single-sample GSEA (ssGSEA) algorithm that calculated the enrichment score and finally determined the infiltrated immune subsets in each sample. Additionally, the immunoactive and immunosuppressive gene signatures were extracted from the tracking tumor immunophenotype (TIP; http://biocc.hrbmu.edu.cn/TIP/).

### Drug Sensitivity Analysis

Genes related to the prognostic model, as well as MYCN and DNMT1 were applied in the drug sensitivity analysis in GSDC with the assistance of GSCALite (http://bioinfo.life.hust.edu.cn/GSCA/#/). The rationale of the drug target prediction integrated many approaches and algorithms according to the extended methods (https://www.cancerrxgene.org/gdsc1000/GDSC1000_WebResources//Home_files/Extended%20Methods.html#17). Drug sensitivity data were obtained from the Genomics of Drug Sensitivity in Cancer (GDSC; https://www.cancerrxgene.org/).

### Statistical Analyses

Nearly all statistical analyses and visualizations were performed using R software v3.6.3. Some of simple visualizations were applied using GraphPad Prism v9.2.0 or GSEA v4.1.0. Unless otherwise specified above, P < 0.05 or FDR<0.25 was considered statistically significant.

## Results

### N6-methyladenosine Erasers in Neuroblastoma

**Figure 1:**
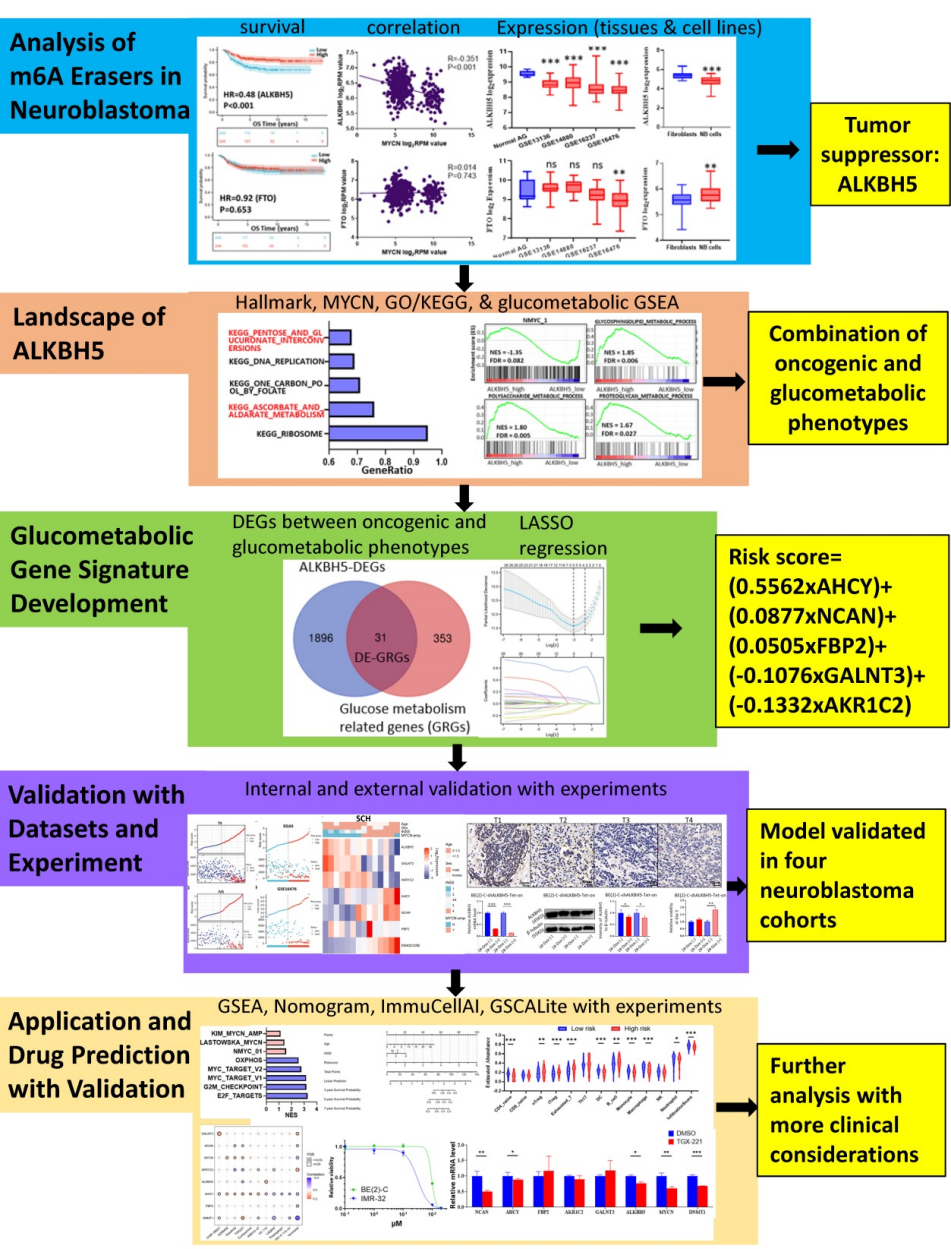
Flowchart of the entire analysis

**Figure 2:**
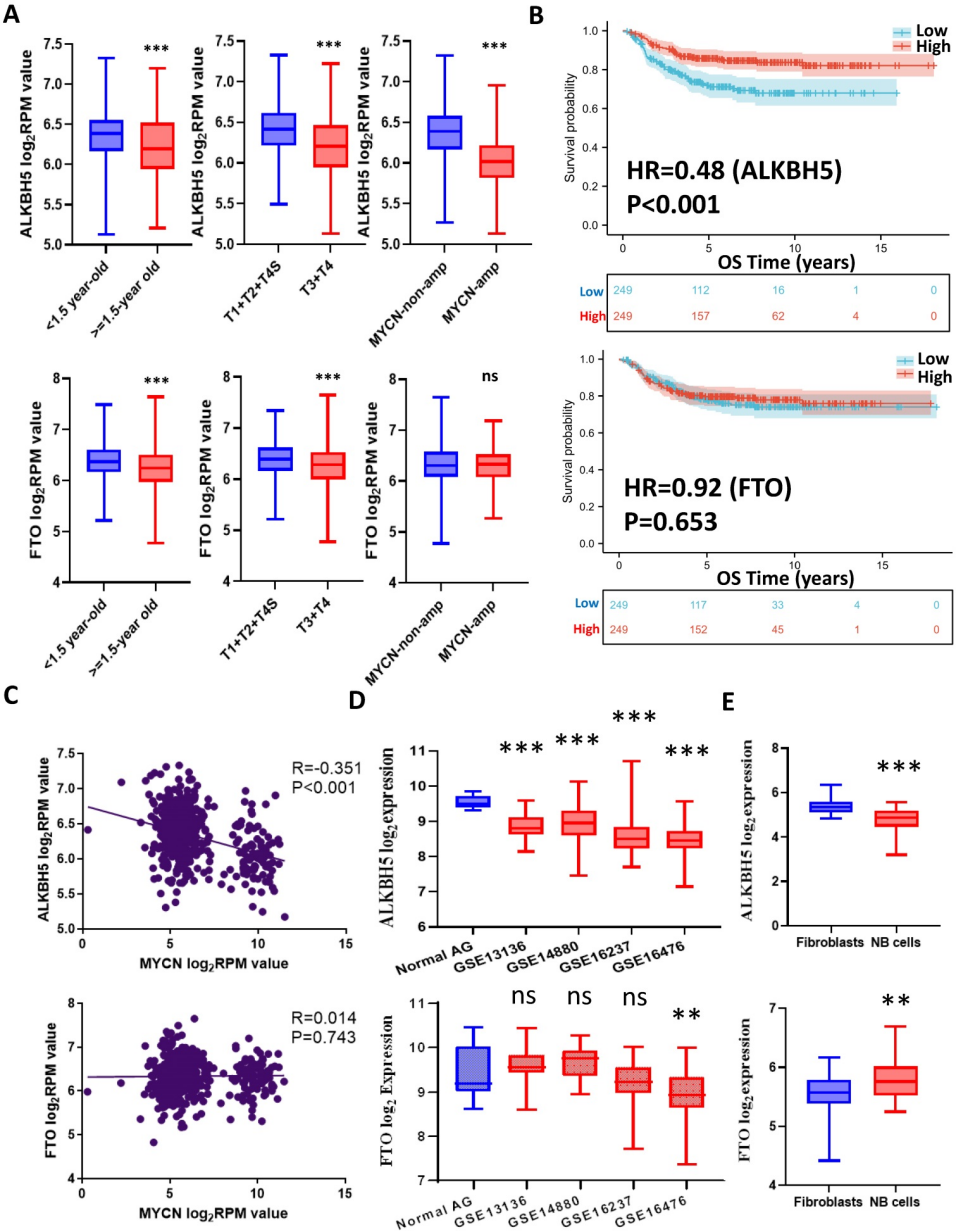
N6-methyladenosine Erasers in Neuroblastoma. (**A**) ALKBH5 (upper panel) and FTO (lower panel) expression in different age, INSS and MYCN status groups. (**B**) Kaplan-Meier curve of the ALKBH5/FTO-high and ALKBH5/FTO-low groups (upper/lower panel). (**C**) Oncogene MYCN correlated with ALKBH5/FTO (upper/lower panel). (**D**) ALKBH5/FTO expression profile (upper/lower panel) across microarray datasets in the GPL570 platform. (**E**) ALKBH5/FTO expression profile (upper/lower panel) from CCLE. HR: hazard ratio. NS: not significant. P<0.05 was shown as *, P<0.01 as ** and P<0.001 as ***.

The workflow of the whole analysis is shown in **Figure 1**. To determine the potential role of m6A erasers in NB, we briefly checked the expression profiles of different groups and the survival data based on the median expression of the m6A erasers ALKBH5 and FTO (**Figure 2A and 2B**) in GSE62564. As an age ≥1.5 years, INSS and MYCN amplification (MYCN-amp) are hazardous indicators in NB patients 2, 3, we analyzed ALKBH5 and FTO expression levels in different age, INSS and MYCN-amp groups and found that ALKBH5 levels were lower in the ≥1.5-year-old group, T3+T4 group and MYCN-amp groups (**Figure 2A** in upper panel). Lower FTO expression was observed in the ≥1.5-year-old group and T3+T4 group, whereas no significant difference was found in the MYCN-amp group (**Figure 2A** in lower panel). In addition, we classified NB patients from the whole dataset into a high-expression group (ALKBH5-high or FTO-high) and a low-expression group (ALKBH5-low or FTO-low) based on the median expression of ALKBH5 or FTO. Kaplan-Meier analysis showed that high ALKBH5 expression in NB tissue samples was positively correlated with better OS (HR=0.48, P<0.001; **Figure 2B** in upper panel), whereas higher FTO expression was not correlated with OS (HR=0.92, P=0.653; **Figure 2B** in lower panel). Furthermore, we analyzed the correlation between MYCN and ALKBH5, as well as MYCN and FTO, showing that ALKBH5 expression was negatively correlated with oncogene MYCN (R=-0.351, P<0.001), whereas there was no significant correlation between MYCN and FTO (**Figure 2C**). Except in GSE62564, we checked other transcriptomic NB data in tissues and cell lines, suggesting that lower expression of ALKBH5 but not FTO in NB compared with normal tissues (**Figure 2D**) and cell lines (**Figure 2E**). Therefore, we focused on ALKBH5 in further analysis.

### Identification and Landscape of ALKBH5

**Figure 3:**
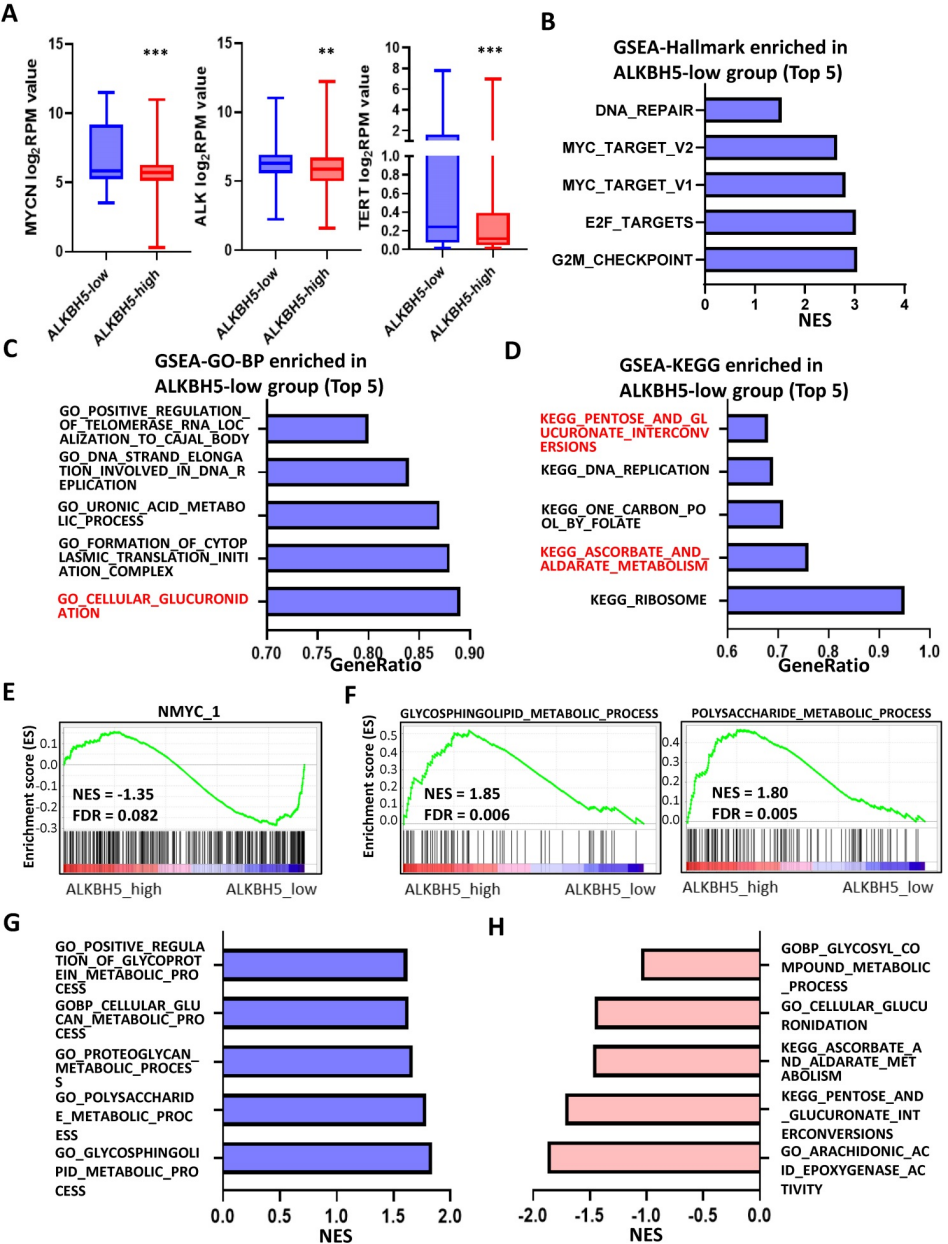
Identification and Landscape of ALKBH5. (**A**) MYCN, ALK and TERT levels in the ALKBH5-high and ALKBH5-low groups. (**B, C, D**) Top 5 Hallmark (B), GO-BP (C) and KEGG (D) gene sets enriched in the ALKBH5-low group obtained by GSEA. (**E**) One representative MYCN-related gene set negatively enriched in ALKBH5-high group. (**F**) Two representative glucometabolic gene sets enriched in the ALKBH5-high group. (**G, H**) Five representative glucometabolic gene sets enriched in the ALKBH5-high (G) and ALKBH5-low (H) group. NES: normalized enrichment score. FDR: false discovery rate. P<0.05 was shown as *, P<0.01 as ** and P<0.001 as ***.

As MYCN and ALK are risk indicators that are routinely examined in Chinese clinics, and TERT are found to be a novel risk factor in NB^16^,we evaluated MYCN,ALK and TERT levels in both the ALKBH5-low and ALKBH5-high groups and found that NB patients with higher ALKBH5 expression had lower MYCN, ALK and TERT levels (**Figures 3A**). GSEA-Hallmark analysis showed that the ALKBH5-low group was enriched with oncogenic phenotypes, such as G2M_checkpoint and E2F_targets (**Figure 3B**). These findings suggest that ALKBH5, as an m6A eraser, might play a protective role in NB tumorigenesis.

Then, we aimed to obtain deeper insight into the biological processes (BPs) of ALKBH5, and GSEA was employed to conduct GO annotation and KEGG pathway enrichment analyses. The top 5 significant GO-BP terms and KEGG pathways were extracted following the functional enrichment analysis of ALKBH5 mRNA, and the terms with red labels within figures show potential relationships with glucose or carbohydrate metabolism (**Figure 3C and 3D**). According to the GO-BP terms ranked by the gene enrichment ratio, the ALKBH5-low group, indicative of NB tumorigenesis, was mainly clustered with the terms “cellular glucuronidation” and “formation of cytoplasmic translation initiation complex” (**Figure 3C**). Among the KEGG terms, it was mainly clustered with “ribosome”, “ascorbate and aldarate metabolism” and “one carbon pool by folate” (**Figure 3D**). The top 5 items in the GO-MF and GO-CC gene signatures were also listed (**Figure S1A and S1B**). Specifically, we checked some of the molecular signatures associated with MYCN (**Figure 3E**) and carbohydrate metabolism (**Figure 3F, 3G, 3H and S1C**). Our GSEA concluded that the ALKBH5-high group was enriched in and characterized by the glucose metabolic processes such as glycosphingolipid and polysaccharide (**Figure 3F, G**). Meanwhile, the ALKBH5-low group was enriched in gene signatures of “NMYC_1” (**Figure 3E**)**, “**arachidonic acid epoxygenase activity” and “glycosyl compound metabolic process” (**Figure 3H**). Based on the effect of ALKBH5 on OS and its possible role in tumor suppression and carbohydrate metabolism, we next aimed to explore the role of glucometabolic dysregulation in NB.

### Identification and Functional Enrichment Analyses of DE-GRGs

**Figure 4:**
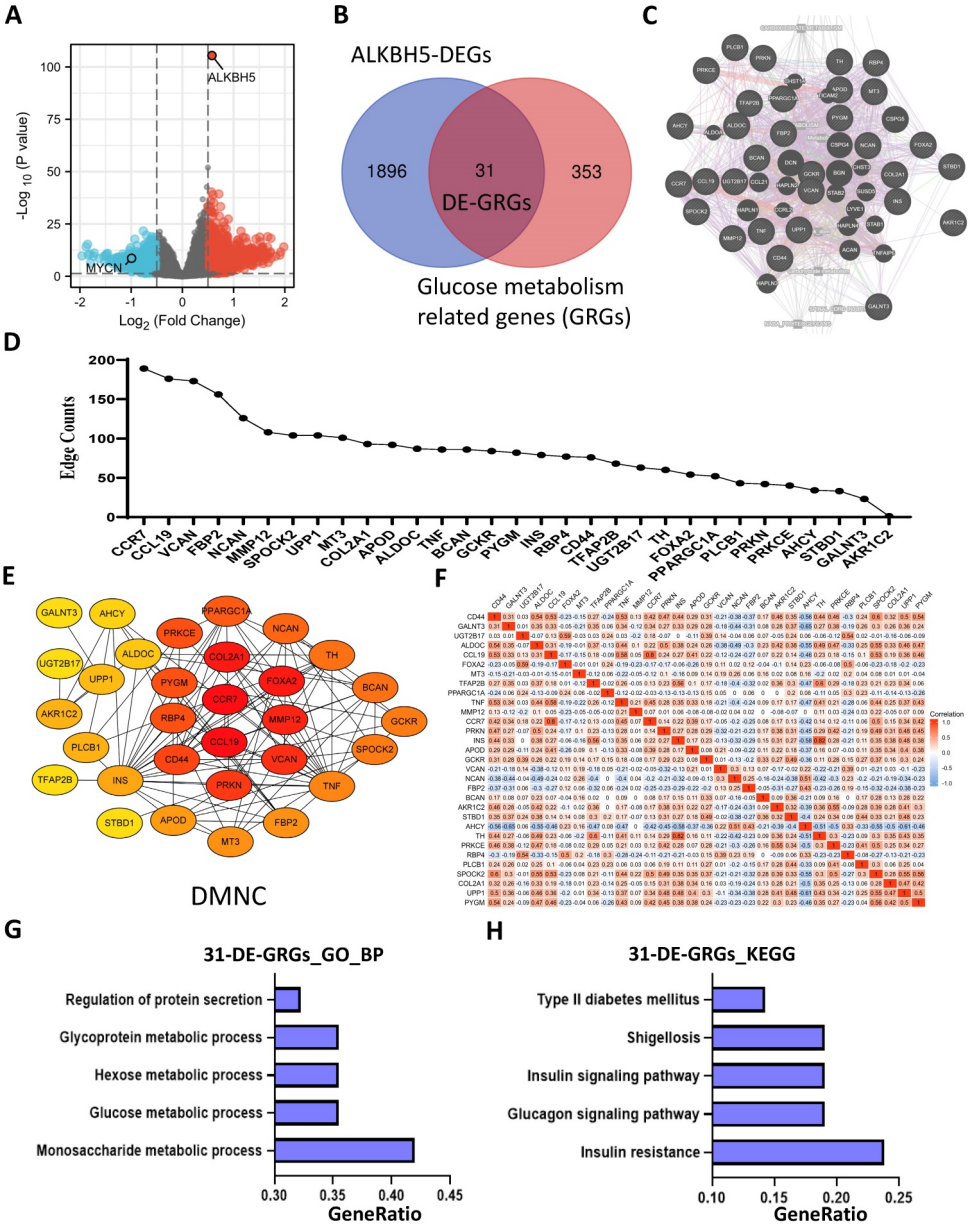
Identification and Functional Enrichment Analyses of DE-GRGs. (**A**) Volcano plot showing differentially expressed genes between the ALKBH5-high and ALKBH5-low groups. (**B**) Venn diagram of 31 overlapping genes (31 DE-GRGs) between ALKBH5-DEGs and GRGs. (**C**) Thirty-one DE-GRGs in the GeneMANIA network. (**D**) Rank of DE-GRGs by edge counts. (**E**) Thirty-one DE-GRGs in the PPI network calculated by DMNC. (**G, H**) Top 5 gene sets functionally enriched by GO-BP (G) annotations and KEGG (H) pathways. DEGs: differential expression genes. GRGs: genes related to glucose metabolism. DE-GRGs: overlapped genes of DEGs and GRGs.

A total of 1927 DEGs were obtained from the ALKBH5-high and ALKBH5-low groups, which were together described as ALKBH5-DEGs, including 1206 upregulated and 721 downregulated genes (**Figure 4A**). Venn analysis was conducted to examine the overlap between the DEG and GRG profiles, demonstrating that there were 31 common DEGs shared between the two groups, which were considered DE-GRGs (**Figure 4B**). The general profile of these 31 DE-GRGs was visualized in **Figure S2A** and** S2B.** To obtain a comprehensive overview of these 31 DE-GRGs, the GeneMANIA and STRING database in combination with CytoHubba was adopted to determine the gene-gene links, PPI pairs and hub genes among the 31 genes. As presented in **Figure 4C**, the 31 DE-GRGs with other interactive genes by GeneMANIA exhibited complicated interactions with each other, and the hub genes were focused on CCR7, CCL19 and VCAN based on edge counts (**Figure 4D**), consistent with the results of STRING database and CytoHubba analysis (“DMNC” approach; **Figure 4E**). Interestingly, INS, TNF and CD44 were considered as the hub genes under the “degree” approach (**Figure S2C**). The correlation matrix also potentially showed the interactions among these 31 DE-GRGs, suggesting that the hub genes might play a tumor suppressive role in NB in somewhat immunogenic manners (**Figure 4F**).

These 31 genes were included in GO and KEGG enrichment analyses to further explore the potential functions of the DE-GRGs. GO-BP analysis revealed that these 31 genes were markedly enriched in the “monosaccharide metabolic process” and “glucose metabolic process” (**Figure 4G**). The top markedly enriched KEGG pathways were “insulin resistance” and “insulin signaling pathway” (**Figure 4H**). Moreover, the GO-MF terms included “carbohydrate binding” and “organic acid binding” (**Figure S2D**), and the GO-CC enriched terms were “Golgi lumen” and “vacuolar lumen” (**Figure S2E**). Taken together, ALKBH5 might act as an anti-oncogene in accordance with immune cells in glucose-dependent pathways.

### Five-gene Prognostic Signature Based on 31 DE-GRGs

**Figure 5:**
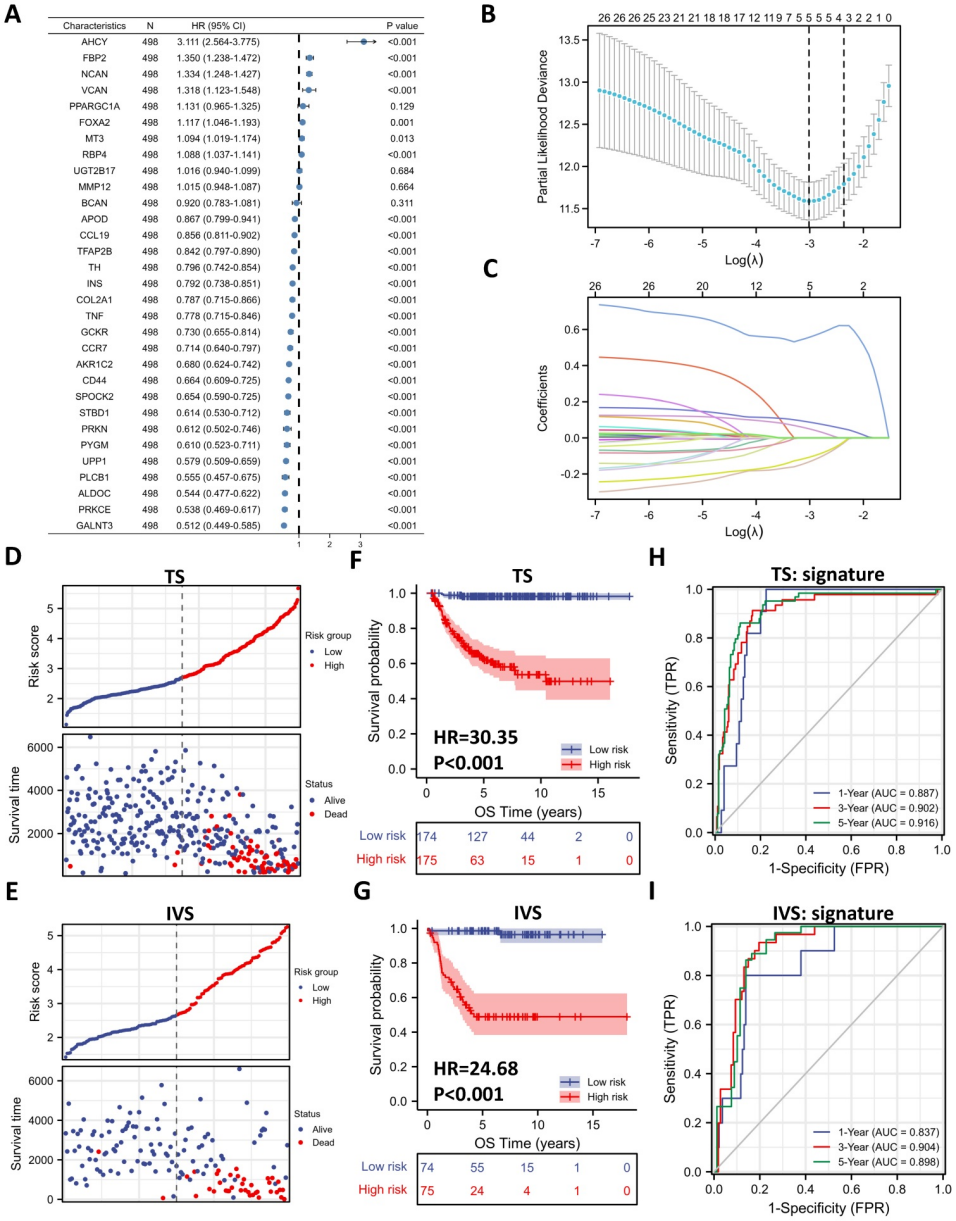
Five-gene Prognostic Signature Based on 31 DE-GRGs. (**A**) Univariate Cox regression showing HR and P values of 31 DE-GRGs. (**B**) LASSO regression screen in TS showing 5 of 27 candidate genes at the least deviance. (**C**) LASSO regression screen in TS showing coefficients of genes at different λ levels. (**D, E**) Risk score and survival distribution in TS (D) and IVS (E). (**F, G**) Kaplan-Meier curve of TS (F) & IVS (G) between the high-risk and low-risk groups determined by the 5-gene prognostic model. (**H, I**) Time-dependent ROC curve in TS (H) and IVS (I) for the 5-gene-based risk score. HR: hazard ratio. TS: training set. IVS: internal validation set. AUC: area under curve.

To investigate the prognostic role of 31 DE-GRGs in NB, we performed a univariate Cox regression analysis of the expression level data in the GSE62564 dataset. The results showed that 27 of the 31 DE-GRGs were significantly associated with OS (P < 0.05), among which 7 genes (AHCY, FBP2, NCAN, etc.) were considered risk genes, with an HR > 1, whereas 20 genes (APOD, CCL19, TFAP2B, etc.) acted as protective genes, with an HR < 1 (**Figure 5A**). In addition to Cox regression analysis, we investigated the correlation between ALKBH5 and 31-DE-GRGs (**Figure S3A**), which showed some similarity with the Cox regression results.

Next, we applied the LASSO algorithm to the 27 genes in TS. Five genes (AHCY, GALNT3, AKR1C2, NCAN, and FBP2) were screened to build the risk signature based on the minimum criteria (**Figures 5B**), and the coefficients obtained from the LASSO algorithm were used to calculate the LRS (**Figure 5C**; LRS=0.5562xAHCY+0.0877xNCAN+0.0505xFBP2+-0.1076xGALNT3+-0.1332xAKR1C2). We also generated correlation matrix among MYCN, ALKBH5 the 5 glucometabolic genes (**Figure S3B**), revealing relatively intense correlations of MYCN with AHCY, NCAN, and FBP2 (positively related) as well as GALNT3, ALKBH5 and AKR1C2 (negatively related).

To further investigate the prognostic role of the 5-gene risk signature, NB patients in both the TS and IVS were categorized into low- and high-risk groups based on the median LRS, and the results indicated that the number of patients who died increased considerably as the risk score increased in both TS and IVS (**Figure 5D and 5E**). Meanwhile, NB patients in the high-risk group showed lower survival in both TS and IVS (**Figure 5F and 5G**). The heatmaps of TS (**Figure S3C**) and IVS (**Figure S3D**) show the glucometabolic signature discriminate the high-risk and low-risk group independent of sex and age. Compared with the low-risk group patients, the high-risk NB patients generally showed higher expression of FBP2, NCAN, and AHCY and lower expression of GALNT3 and AKR1C2 in both TS and IVS (**Figures S3C and S3D**). In addition, the NB patients in the high-risk group were older and exhibited higher MYCN expression (**Figure S3C and S3D**).

ROC curve analyses of the prognostic risk score at 1, 3, and 5 years were performed to test the predictive efficiency of the risk signature. The results showed that the risk score exhibited high accuracy that the area under curve (AUC) of all results in the ROC curves was > 0.800) in distinguishing the OS of NB in both TS and IVS (**Figure 5H and 5I**). We also calculated AUC in other independent risk factors such as MYCN, showing that the 5-gene signature had the highest AUC compared with other independent risk indicators (**Figure S3E and S3F**). To conclude, the newly established prognostic model performs well in GSE62564.

### External Validation of the Gene Signature with Public Datasets

**Figure 6:**
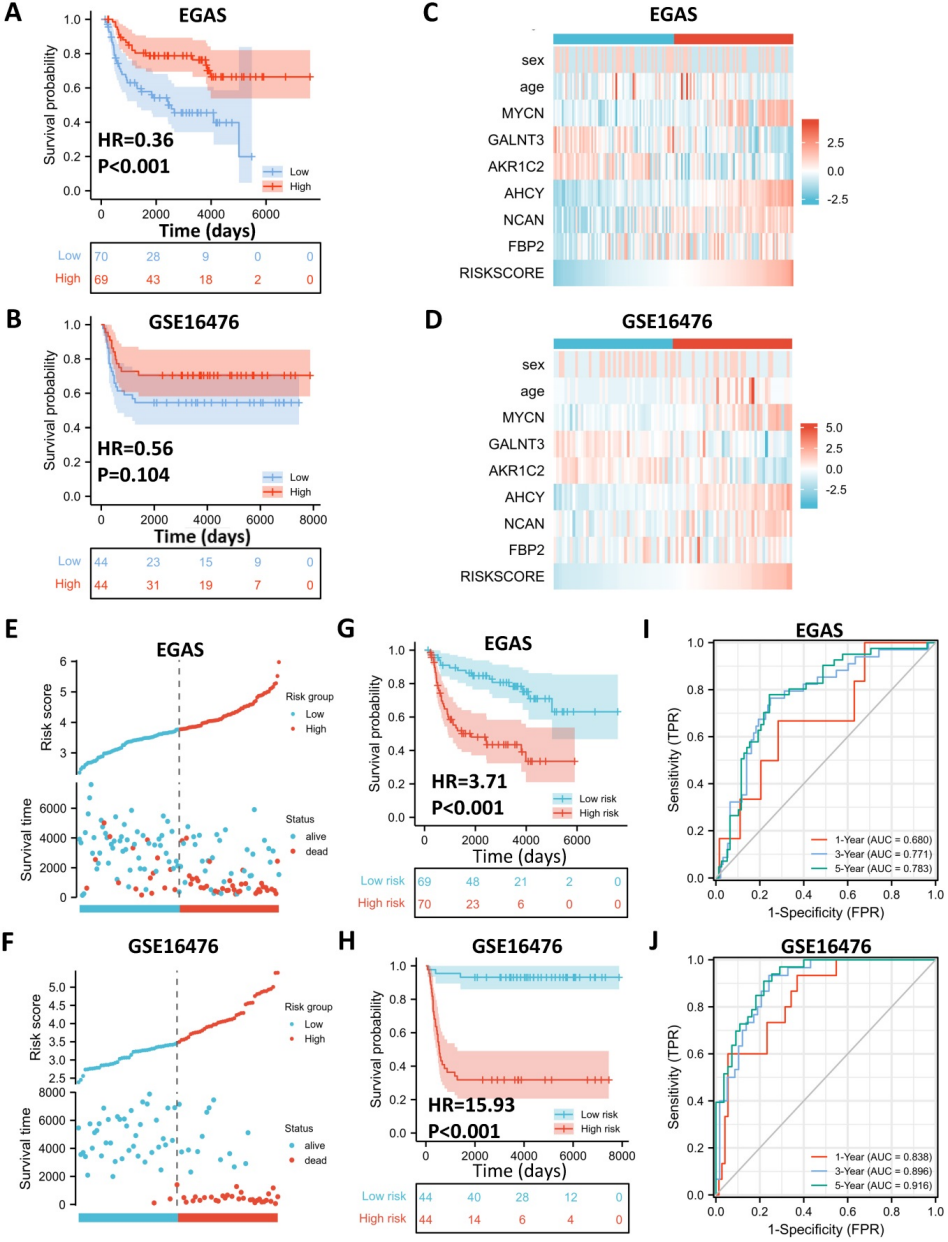
External Validation of the Gene Signature with Public Datasets. (**A, B**) Kaplan-Meier curve of EGAS00001001308 (EGAS) (A) and GSE16476 (B) between the ALKBH5-high and ALKBH5-low groups. (**C, D**) Heatmap showing the profile of clinical characteristics and the 5-gene model in EGAS (C) and GSE16476 (D). (**E, F**) Risk plot of EGAS (E) and GSE16476 (F). (**G, H**) Kaplan-Meier curve of EGAS, GSE16476 between the high-risk and low-risk groups based on the median LRS. (**I, J**) Time-dependent ROC curve in EGAS (I) and GSE16476 (J) for the 5-gene-based risk score. HR: hazard ratio. AUC: area under curve.

To determine whether the 5-gene model can be applied in other NB cohorts, we selected 2 external datasets, consisting of one RNA-seq dataset, EGAS00001001308 (abbreviated as EGAS later), and one microarray dataset, GSE16476, to conduct subsequent validation. ALKBH5 still might play a protective role in NB tumorigenesis in both the RNA-seq (HR=0.36) and microarray (HR=0.56) datasets, although no significant difference was found in GSE16476 (**Figure 6A and 6B**). By applying LASSO risk coefficients to these 2 external datasets, we found that the 5-gene prognostic model performed well according to expression profile (**Figure 6C and 6D**), risk plot (**Figure 6E and 6F**), Kaplan-Meier curve (**Figure 6G and 6H**), and ROC curve analyses (**Figure 6I and 6J**). Moreover, we demonstrated gene expression of ALKBH5 and 5 glucometabolic genes in different INSS groups and MYCN-amp groups (**Figure S4A and S4B**) and the correlation matrix was also shown to view the potential interactions (**Figure S4C and Figure S4D**). In brief, the glucometabolic gene model is applicable in external datasets.

### ALKBH5-enriched Gene Signature in the SCH Cohort and NB Cell Line Validation

**Figure 7:**
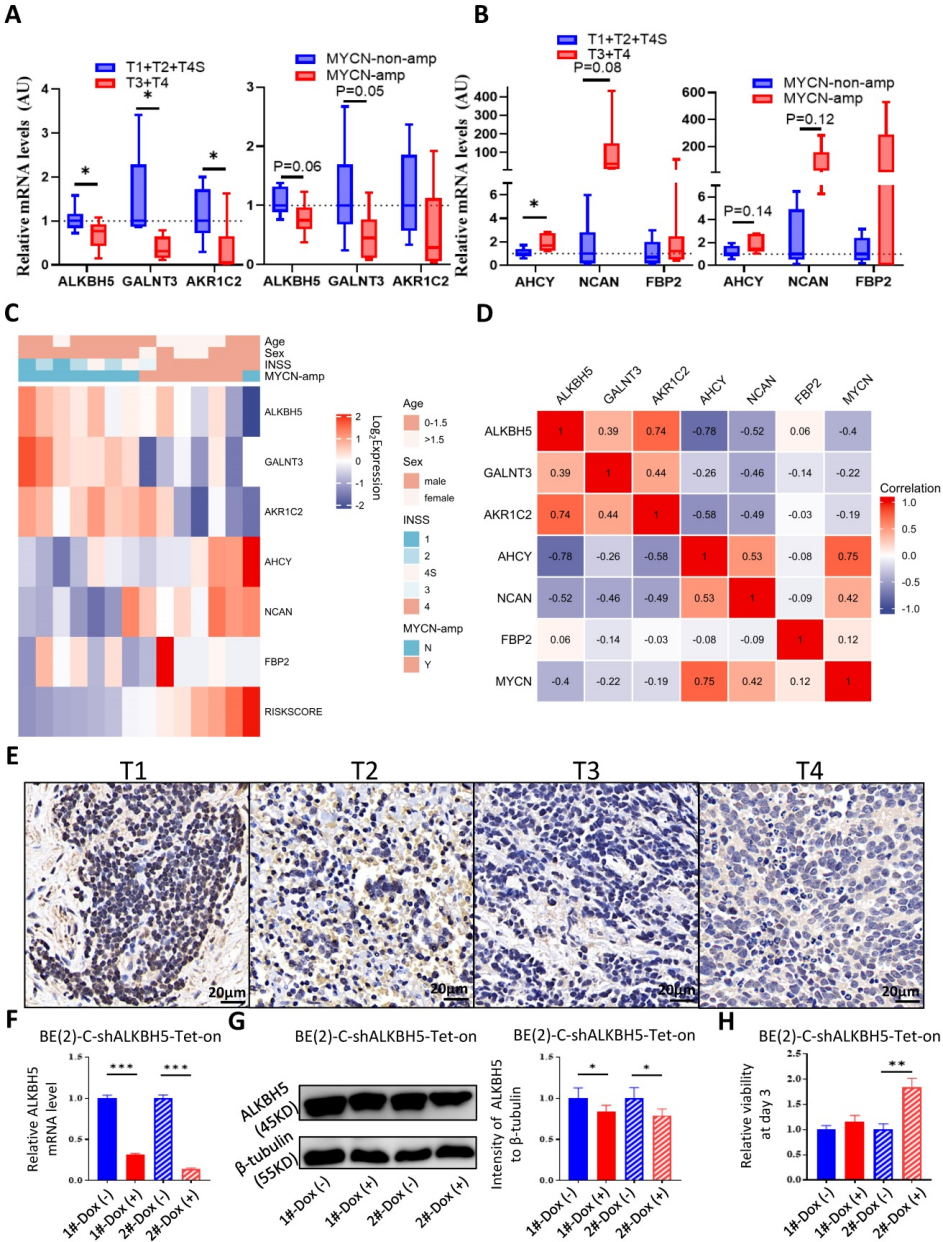
External Validation of the Gene Signature with the SCH Cohort and Biological Experiments. (**A, B**) Transcriptional level of tumor suppressor genes (A) and oncogenes (B) in the model among NB tissue samples based on INSS (left panel) and MYCN amplification status (right panel) from SCH. (**C**) Heatmap showing the profile of clinical characteristics and the 5-gene model in the SCH cohort. (**D**) Matrix showing gene-gene correlation value among MYCN, ALKBH5 and other 5 genes in the model in the SCH cohort. (**E**) Representative ALKBH5 immunostaining images among NB tissues in different stages. (**F**) Transcriptional level of ALKBH5 in BE(2)-C-shALKBH5 Tet-on cells. (**G**) Representative immunoblotting images and quantification of ALKBH5 protein expression in BE(2)-C-shALKBH5 Tet-on cells. (**H**) Relative viability of BE(2)-C-shALKBH5 Tet-on cells. 1# and 2# represented different shRNA targets. Dox, doxycycline. NS: not significant. P<0.05 was shown as *, P<0.01 as ** and P<0.001 as ***.

After external dataset validation, we checked the accuracy of the glucometabolic signature in NB tissue samples from SCH. Based on INSS, we compared the transcriptional levels of tumor suppressor genes, including ALKBH5, GALNT3 and AKR1C2, and oncogenes, including AHCY, NCAN and FBP2, between the T1+T2+T4S cohort and the T3+T4 cohort (n=7 vs. n=7) by Q-RT-PCR, revealing lower levels of suppressor genes (**Figure 7A**; left panel) and higher levels of oncogenes (**Figure 7B**; left panel) in the T3+T4 cohort. Similarly, compared with the NB patients in the MYCN-non-amp group, tumor suppressor gene expression was relatively lower in the MYCN-amp group (n=8 vs. n=6; **Figure 7A**; right panel), whereas oncogene expression was relatively higher in the MYCN-amp group (**Figure 7B**; right panel). Some of the differences were not considered statistically significant due to the small number of samples and low basal expression of FBP2. Moreover, the heatmap was drawn to visualize the profile of gene expression and clinical characteristics in the SCH cohort (**Figure 7C**), revealing a high similarity with GSE62564 (**Figure S3C and S3D**), EGAS (**Figure 6C**) and GSE16476 (**Figure 6D**)**.** Meanwhile, the gene-gene correlation matrix in the SCH cohort was visualized (**Figure 7D**) and showed consistency with GSE62564 (**Figure S3B**) and EGAS (**Figure S4C**). Moreover, we checked ALKBH5 expression in protein level and showed a decreased tendency from T1 to T4 (**Figure 7E**).

To validate the function of ALKBH5 as a tumor suppressor in cells, we construct BE(2)-C-shALKBH5-Tet-on STCs and found that 1μg/mL of Dox was capable to induce ALKBH5 knockdown in both mRNA level (**Figure 7F**) and protein level (**Figure 7G**). Genetic attenuation of ALKBH5 slightly promoted cell growth at day 3 of cell culture (**Figure 7H**). The results in NB cells showed that ALKBH5 functioned as a suppressor somehow. Taken together, we used our NB tissue samples and cells to validate the capability of glucometabolic gene signature and the potential tumor suppressive role of ALKBH5.

### Utility of the Glucometabolic Model in Assessing Biological and Clinical Status of Neuroblastoma

**Figure 8:**
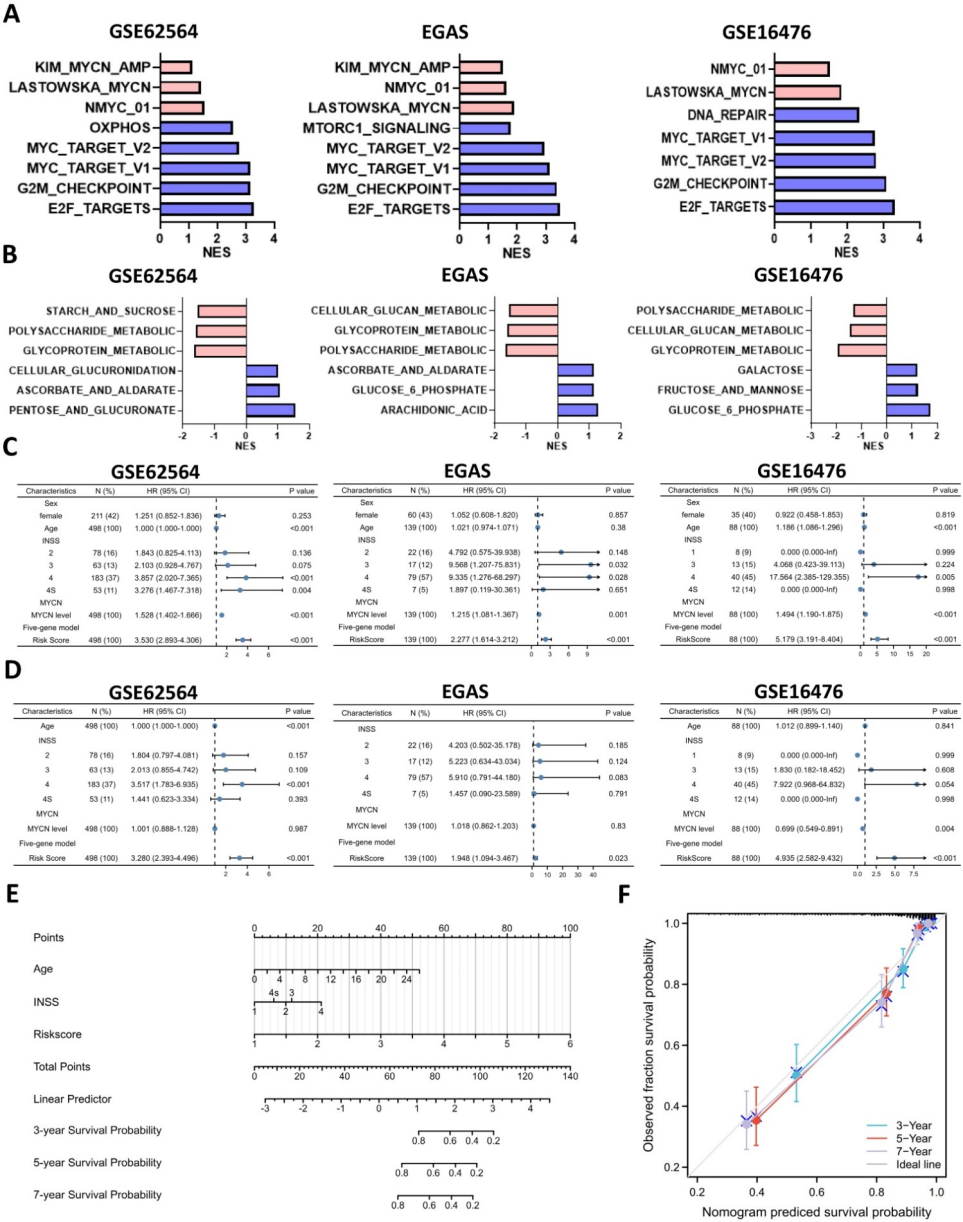
Utility of the Glucometabolic Model in Assessing Biological and Clinical Status of Neuroblastoma. (**A**) Top 5 Hallmark gene sets and MYCN-related gene sets enriched in the high-risk group obtained by GSEA in GSE62564, EGAS and GSE16476. (**B**) Representative three glucometabolic gene sets enriched in the high-risk group (blue columns) and low-risk group (red columns) obtained by GSEA in GSE62564, EGAS and GSE16476. (**C, D**) Univariate (C) and multivariate (D) Cox regression for sex, age, INSS, MYCN level and risk score by a 5-gene model in GSE62564, EGAS and GSE16476. (**E**) Nomogram for clinical practitioners in GSE62564. (**F**) Calibration plot (100 subjects per group, resampling time = 1000) for the nomogram in GSE62564.

To better understand the biological characteristics of NB in the newly developed prognostic model, we used the newly constructed model to classify NB patients to the high risk and low risk group based on the median LRS among GSE62564, EGAS and GSE16476. Comprehensive GSEA in 3 different cohorts was performed by applying Hallmark gene sets & MYCN-related gene sets (**Figure 8A**) and glucometabolic gene sets (**Figure 8B**). The high-risk group was enriched in positive normalized enrichment score (NES), suggesting that NB patients in the high-risk group were characterized with oncogenic (blue columns in **Figure 8A**) and MYCN-related (red columns in **Figure 8A**) phenotypes. Moreover, the NB samples from the high-risk group was positively related to glucometabolic GO/KEGG terms of “pentose and glucuronate” and “glucose-6-phosphate” (blue columns in **Figure 8B**), and negatively related to “glycoprotein” and “polysaccharide” (red columns in **Figure 8B**), showing somewhat similarity with GSEA in ALKBH5 (**Figure 3B-H and S1A-C**).

Clinical characteristics of NB including age and sex, as well as risk factors, including INSS and MYCN levels in the model were also investigated. We performed univariate and multivariate Cox regression analyses of GSE62564, EGAS and GSE16476 dataset to determine whether the risk signature was an independent prognostic indicator among other clinical characteristics. Univariate Cox regression showed that sex (P>0.05) and age (HR value close to 1) were not risk factors, in contrast to INSS stage 4, MYCN level and the risk score of the 5-gene model (HR>1, P<0.05; **Figures 8C**). The results of multivariate Cox regression analyses presented in **Figure 8D** indicated that MYCN level (P>0.05 in GSE62564 and EGAS, HR<1 in GSE16476) were not risk factors; however, the risk score of the 5-gene model (HR=3.28, P<0.001 in GSE62564; HR=1.95, P=0.023 in EGAS; HR=4.94, P<0.001 in GSE16476) was still a risk indicator, similar to the well-established INSS stage 4 (HR=3.517, P<0.001; HR=5.91, P=0.083; HR=7.922, P=0.054), and even a better indicator with statistical difference in three different datasets. We further constructed a nomogram of clinical prediction in combination with age, INSS and the risk score of the 5-gene model to provide a quantitative method for clinical practitioners to predict the probability of the 3-, 5‐ and 7‐year OS of NB patients. In the prediction nomogram (**Figure 8E**), each patient received some points for each prognostic parameter, and a greater number of total points suggested an inferior prognosis, in combination with a well-performed calibration plot (**Figure 8F**).

### Immune Landscape of Neuroblastoma Patients and Potential Targets

**Figure 9:**
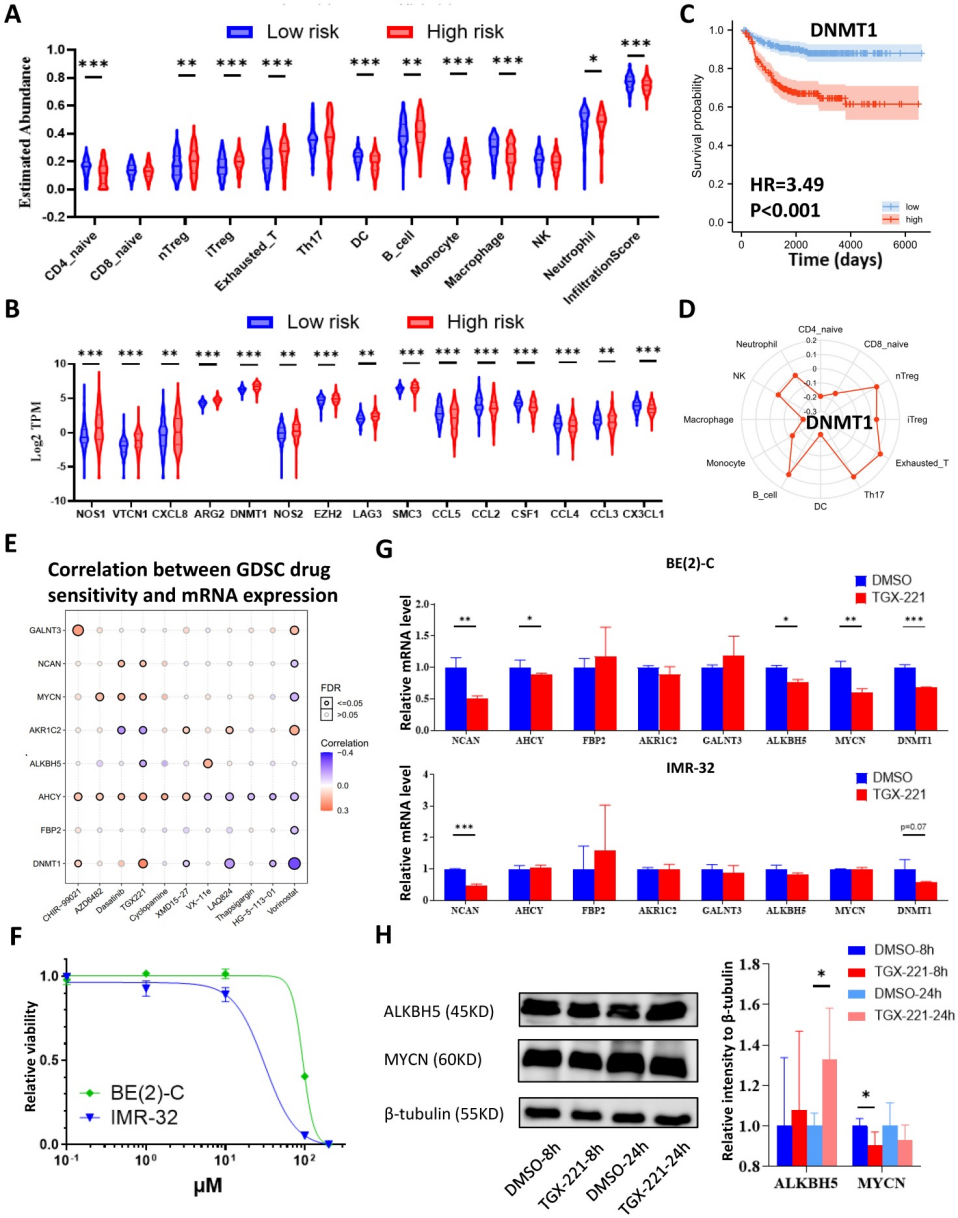
Immune Landscape of Neuroblastoma Patients and Potential Targets. (**A, B**) Boxplot visualizing the difference of leukocyte infiltration (A) and immune-related markers (B) among different risk groups based on the 5-gene model from GSE62564. (**C**) Kaplan-Meier curve of GSE62564 between the DNMT1-high and DNMT1-low groups. (**D**) Radar map showing relationship between immune cells and DNMT1. (**E**) Matrix showing correlation between GDSC drug sensitivity and mRNA expression (which includes MYCN, ALKBH5, DNMT1 and 5 genes in the prognostic model). (**F**) Dosage curve of TGX-221 in BE(2)-C and IMR32 cells. (**G**) Transcriptional change of MYCN, ALKBH5, DNMT1 and 5 genes in the model after 8 hours of TGX-221 treatment. (**H**) Representative immunoblotting image and quantification of ALKBH5 and MYCN after 8 and 24 hours of TGX-221 treatment. nTregs: natural T regulatory cells. iTregs: inducible T regulatory cells. DCs: dendritic cells. HR: hazard ratio. NS: not significant. P<0.05 was shown as *, P<0.01 as ** and P<0.001 as ***.

Analyses of glucometabolic hub genes showed that CCR7, CCL19, VCAN, NCAN, FBP2, MMP12, CD44, INS, TNF, etc. had the great number of edge counts (**Figure 4C-E and S2C**), and most of these genes were indicative to immune response. Therefore, we performed ssGSEA with the help of ImmuoCellAI online tool to show immune cell infiltration in NB patients (GSE62564 as the representative dataset, n=498). As shown in **Figure 9A,** the immunosuppressive natural T regulatory cells (nTregs), inducible T regulatory cells (iTregs) and exhausted T cells were increased and the immunoactive monocytes, macrophages and dendritic cells (DCs) were reduced in the high-risk NB subtype (classified by 5-gene glucometabolic signature). By focusing immunosuppressive T cells and monocytes (as well as monocyte-differentiated macrophages), we downloaded the gene list of anti-inflammatory cytokines related to immunosuppressive T cells and pro-inflammatory cytokines related to monocytes, and visualized the increased immunosuppressive markers and the decreased immunoactive markers showing that NOS1 had the most dramatic increment and CCL5 had the most dramatic decline (**Figure 9B**). Immunosuppressive marker NOS1 that was positively correlated with iTregs & exhausted T cells and negatively correlated with macrophages & DCs (**Figure S5A**), and immunoactive marker CCL5 that was positively correlated with macrophages & DCs and negatively correlated with iTregs & exhausted T cells (**Figure S5B**). Kaplan-Meier analysis of NOS1 and CCL5 (**Figure S5C**) showed that patients with higher NOS1 expression had poorer OS (left panel) whereas higher CCL5 expression indicated better OS (right panel). Subsequently, we explored all correlations between NOS1/CCL5 and all immune cells (**Figure S5D**) and found that NOS1 might also play an immunosuppressive role in CD4 & CD8 naïve T cells and CD4^+^ Th17 cells (left panel) and CCL5 might also be potential in boosting CD4 & CD8 naïve T cells and CD4^+^ Th17 cells (right panel).

We further aimed to conduct a drug sensitivity analysis using the GDSC database with the help of GSCALite online tool. This analysis was based on basal gene expression of cell lines and a very low mRNA level of NOS1 and CCL5 was tested in NB cell lines (data not shown). Therefore, we investigated the immunosuppressive indicator DNMT1 which had the highest mRNA level in NB cell lines and might contribute more to poor prognosis (**Figure 9C**; HR=3.49) compared with NOS1 (**Figure S5C**; HR=1.96). DNMT1 was positively correlated with nTregs & exhausted T cells and negatively correlated with macrophages and DCs (**Figure S5E**). Moreover, DNMT1 might play an immunosuppressive role in CD4 and CD8 naïve T cells (**Figure 9D**), which was similar to NOS1.

Then we selected MYCN, ALKBH5, DNMT1 and 5 genes in the model to perform a drug sensitivity screening and revealed that TGX221 was the most potential drug as its positive sensitivity with MYCN, NCAN, AHCY & DNMT1 and negative sensitivity with ALKBH5 and AKR1C2 (**Figure 9E**). Next, we tested TGX-221 in MYCN-amplified BE(2)-C and IMR-32 cell lines and found IC50 of TGX-221 was 94.29 μM in BE(2)-C and 31.24 μM in IMR-32 (**Figure 9F**). Therefore, we treated the NB cells with 100 μM TGX-221 for 8 hours and found that a declined mRNA level in NCAN, AHCY, MYCN & DNMT1 in BE(2)-C and NCAN & DNMT1 in IMR-32 (**Figure 9G**). Immunoblotting analysis showed that TGX-221 increased ALKBH5 expression after 24 hours and slightly decreased MYCN expression after 8 hours (**Figure 9H**). Altogether, these results indicate that increased immunosuppressive T cells and decreased monocytes & DCs are associated with higher levels of NOS1 & DNMT1 and lower level of CCL5 in the NB high-risk group, and TGX221 is a potential agent in inhibiting the oncogenic and glucose-dysregulated pathway.

## Discussion

Despite multimodal therapeutic strategies, COG-defined high-risk neuroblastoma with < 50% OS in 5 years is a critical issue to be focused on. The MYCN (or N-MYC) oncogene is an absolute determinant defining the high-risk NB 2, 3. The specifically highly expressed MYCN in NB provokes the elevated expression of a set of genes such as PHOX2B, ASCL1, GATA3, ISL-1, HAND2, and TBX2, referred to as the core regulatory circuit (CRC), which is closely associated with super enhancers and epigenetic abnormalities 12, 17. m6A RNA modification is an epigenetic process that has been extensively studied in many types of cancer in recent years 7, 8. However, far fewer of the m6A studies have been conducted in NB than in other types of cancer. The m6A writers METTL3 10 and METTL14 18 have been demonstrated to play an oncogenic role in clinical samples; nonetheless, few studies regarding m6A erasers have been conducted.

Our integrative bioinformatic analysis suggested that the m6A eraser ALKBH5 might play a tumor-suppressive role in NB compared with another m6A eraser FTO. Moreover, in contrast to FTO, ALKBH5 presented higher expression in normal AG tissues and fibroblast cell lines. Subsequent analyses indicated that ALKBH5 might play a regulatory role in glucometabolic processes, and related glucometabolic genes were screened to build a new prognostic model. The 5-gene signature (AHCY, NCAN, FBP2, GALNT3, and AKR1C2) in the model showed good clinical prediction potency in TS, IVS and EVS, and the transcriptional profile was validated by the use of NB samples from our own hospital. Estimation of immune cell subtypes showed an increase of immunosuppressive T cells and decrease of monocytes, macrophages and DCs. At last, drug sensitivity screen and validation showed that TGX221, a kind of PI3K-β inhibitor, might be the most potential drug in the glucose-dysregulated pathway. Altogether, our work is the first study to perform a combined analysis of the m6A eraser ALKBH5 and carbohydrate metabolism in NB.

Excepts for the demethylase activity of ALKBH5 in m6A process, ALKBH5 also represents alpha ketoglutarate dependent dioxygenase 19, which is involved in the biological reaction of ketoglutarate to succinate in tricarboxylic cycle (TCA cycle). In addition to TCA cycle, ketoglutarate acts as the substrates in amino acid metabolic processes, such as glutamate, known as neurotransmitter. We hypothesize that less TCA cycles occur in the high-risk NB patients with lower ALKBH5 expression in parallel to more aerobic glycolysis (Warburg effect), mimicking the oncogenic phenotypes of the ALKBH5-low NB patients. In combination with our all GSEA results in glucose metabolism, they suggest that ALKBH5 is generally involved in polysaccharide and glycoprotein metabolic processes and lack of ALKBH5 is generally associated with glucose-6-phosphate and ascorbate & aldarate metabolism. Sohretoglu et al. summarized that natural polysaccharide might be an anti-cancer agent 20. However, there are a huge amount of glycoproteins including “cancer feeders” 21 and “cancer killers” [22]so that it is hard to elucidate the relationship between ALKBH5 and glycoproteins.

Among the oncogenes in the model, AHCY represents adenosylhomocysteinase, which is indicated to catalyze the transformation of adenosylhomocysteine to adenosine and L-homocysteine. In our GO/KEGG enrichment analysis, AHCY was included in the “glycosyl compound metabolic process” gene set, suggesting that AHCY might link glucose with adenosine or homocysteine. Interestingly, AHCY is often selected in other LASSO models 23, 24, and its molecular function in viability has been proven in MYCN-amp NB cell lines 25 with a median dependency score <-0.5 in NB cell lines according to the Depmap database (https://depmap.org/portal/). NCAN, also known as neurocan, is considered to be a type of proteoglycan involved in the modulation of cell adhesion and migration. NCAN was associated with “glycosaminoglycan metabolic process”, “proteoglycan metabolic process” and “glycoprotein metabolic process”, suggesting that NCAN might serve as a type of proteoglycan and participate in glycoprotein metabolic processes. Its function of viability was also proven in a previous report 26, with a median dependency score <0 in NB cell lines. FBP2 represents fructose-bisphosphatase 2, which is a gluconeogenesis enzyme involved in glycolysis, with a median dependency score <0 in NB cell lines. There are no reports concerning FBP2 in NB, possibly due to their low expression in NB tissue samples.

Among tumor suppressor genes in the model, GALNT3 represents polypeptide N-acetylgalactosaminyltransferase 3, participating in the processes of O-linked oligosaccharide biosynthesis and glycoprotein metabolic processes. GALNT3 is reported to play a tumor-suppressive role in familial tumoral calcinosis 27 and lung cancer 28 and shows a median dependency score >0 in NB cell lines; however, there is no available study regarding GALNT3 in NB. AKR1C2 represents aldo-keto reductase family 1 member C2, which catalyzes the conversion of aldehydes and ketones to their corresponding alcohols and is associated with “glycosyl compound metabolic process”. Interestingly, AKR1C2 has a median dependency score <0 in NB cell lines, in contrast to our findings in tissue datasets. However, AKR1C2 is generally considered to act as a tumor suppressor according to several cancer reports 29, 30. Similar to GALNT3, few studies have examined AKR1C2 in NB.

In addition to GALNT3 and AKR1C2, we performed some biological experiments concerning ALKBH5. The median dependency score of ALKBH5 in NB cell lines is about -0.1. It should be mentioned that knockdown of ALKBH5 might slightly inhibit the long-term NB cell growth (data not shown) that was consistent with the recent reports in glioma 31, 32. Moreover, TGX-221 treatment slightly inhibited ALKBH5 expression in early time, which was contradictory to the main results that we found. The tumor growth in vitro controlled by genes and the tumor growth that affects patients in vivo are not completely consistent. For instance, GATA3 is commonly regarded as an oncogene 17, 33 with the median dependency score <-0.5 in NB cell lines, however, it is positively correlated with good prognosis in GSE62564 (HR=0.68). Moreover, some reports 34, 35 indicated that GATA3 might act as a tumor suppressor. Therefore, these discrepancies remain to be elucidated in the future studies.

A large number of reports have indicated a correlation between glucometabolic processes and immune response. In our model, we showed the most dramatic change in immunosuppressive NOS1 and immunoactive CCL5, as well as the highest expression in immunosuppressive DNMT1. Except for NOS1, DNMT1 and CCL5, the hub genes CCL19 and CCR7 in DE-GRGs are compose of the CCL19/CCL21-CCR7 axis that exerts both immune response and tumor proliferation 36. The ALKBH5-high (low-risk) group showed an increase of CCL19-CCR7 expression, suggesting that a more dominant role in inflammatory response in NB patients. The immune response of DCs was reported to be related with CCL19-CCR7 axis 36, consistent with an increase of DCs in the low-risk group. Interestingly, there were a few studies concerning AHCY^37^ /NCAN^38^ /FBP2 /GALNT3^39^ /AKR1C2 40 and T regulatory cells/macrophages, but most of these studies contradicted with our results in immunological manners, possibly because these studies were performed in different types of tumor.

The ultimate goal of NB research is to find an effective agent to inhibit NB tumor growth and improve OS in the high-risk patients. Therefore, the drug sensitivity screen in our model showed that TGX-221 was potential compound in finetuning dysregulation of glucose metabolism. TGX-221 was a PI3K-β inhibitor and its derivatives were administered in ongoing or completed clinical trials (e.g., NCT03213678, phase II). We performed some experiments with TGX-221 and found its suppressive role in MYCN that was consistent with previous reports of PI3K inhibitor 41. NCAN and DNMT1 were commonly downregulated by TGX-221 in two NB cell lines, suggesting they were potentially involved in PI3K signaling in NB. Few NB reports have been found about the issue and it will be investigated in our future studies.

Several limitations of this work should be noted. We actually tried to perform more external validations and further consolidate our findings; nonetheless, there were a few datasets with complete survival data, and the expression levels of 5 genes could not be extracted or downloaded in every open-access dataset. In addition, we did not extract single-cell or single-nucleus transcriptomic datasets to validate immune cell subtypes in different NB subtypes. This study mainly included analyses of NB tissue datasets with a brief validation, however, a specific cause-effect relationship of ALKBH5 and the glucometabolic signature in NB cells or immune cells remains to be elucidated by further biological experiments.

In conclusion, our integrative study revealed a prognostic gene signature composed of 5 glucometabolic genes correlated with the m6A eraser ALKBH5, which is of considerable significance for the OS of NB patients and may serve as novel biomarkers for the prognosis of patients and even therapeutic targets in the future.

## Supplementary Material

Supplementary tables.

Supplementary figures.
