# Supplementary material for: Identification and Characterization of a Glucometabolic Prognostic Gene Signature in Neuroblastoma based on N6-methyladenosine Eraser ALKBH5
Source: J Cancer. 2022 Mar 28;13(7):2105–25. doi: 10.7150/jca.69408 (PMC9066222; doi:10.7150/jca.69408)
Supplement: Supplementary file 1 — Supplementary tables. [file jcav13p2105s1.pdf]

Supplementary Table 1

KEGG/GO	Description	GeneRatio	BgRatio	pvalue	p.adjust	qvalue	NCBI Entrez Gene_id
KEGG	Galactose metabolism	22/2798	31/7405	0.000177902	0.000496716	0.000218785	B4GALT1/PFKL/PGM1/HK2/PFKP/GCK/HK1/GALK1/UGP2/GAA/PGM2/LALBA/GLA/GALE/PFKM/G6PC/B4GALT2/LCT/GALM/HKDC1/AKR1B10/G6PC2
KEGG	Fructose and mannose metabolism	23/2798	33/7405	0.000195475	0.000540431	0.00023804	PFKFB3/PFKL/ALDOA/HK2/ALDOC/PFKP/TPI1/HK1/ALDOB/FBP1/TSTA3/SORD/PFKM/TKFC/PMM1/PFKFB1/PMM2/FBP2/MPI/GMPPB/GMPPA/HKDC1/AKR1B10
KEGG	Starch and sucrose metabolism	19/2798	36/7405	0.047467193	0.082121155	0.036171319	GBE1/PGM1/HK2/GPI/GYS1/GCK/HK1/PYGM/UGP2/GAA/PGM2/AGL/G6PC/PYGB/GYS2/PYGL/ENPP1/HKDC1/G6PC2
GO	glucose metabolic process	124/4318	204/18670	6.42E-31	7.50E-29	2.63E-29	ATF3/TNF/PHLDA2/SIK1/IRS2/PGK1/PDK1/PFKL/ALDOA/ENO2/PGM1/HK2/ALDOC/GPI/ENO1/PFKP/TPI1/GAPDH/GCK/IGFBP3/HK1/PDK3/ALDOB/ENO3/GAPDHS/GAA/PKLR/RORA/PGM2/BRS3/TKTL1/PGAM2/SLC37A4/PPARGC1A/SLC25A1/FBP1/PCK1/FABP5/PPARD/TP53/PPP1CA/PTPN2/LEPR/DDB1/MAPK14/PMAIP1/ADIPOQ/SORBS1/SLC25A10/GPD2/LEP/DLAT/MDH2/IGFBP4/NPY1R/TFF3/DCXR/SORD/AKT1/ACTN3/AKT2/IGF1/PC/PFKM/APOD/OAS1/BPGM/IRS1/INPPL1/SRC/GHRL/PIK3CA/DYRK2/ATF4/SERP1/GSK3B/GOT1/G6PD/GOT2/SELENOS/PDK4/GNMT/RBP4/GCKR/G6PC/ARPP19/MLYCD/GPD1/MDH1/PDHB/PPARA/CPT1A/PDHA1/SLC25A11/COX11/SLC25A12/PKM/PFKFB1/PGAM1/FBP2/AKR1A1/SLC25A13/ONECUT1/NFE2L1/ADIPOR1/RGN/MST1/ENPP1/GALM/PDHA2/PGK2/PHKG2/MTOR/POMC/SIRT1/IGF2/PDK2/PRKN/TFAP2B/FOXA2/INS/PDX1/FOXO1/G6PC2
GO	hexose metabolic process	139/4318	244/18670	2.58E-30	2.76E-28	9.66E-29	ATF3/TNF/PHLDA2/PFKFB3/B4GALT1/FUT4/SIK1/IRS2/PGK1/PDK1/PFKL/ALDOA/ENO2/PGM1/HK2/ALDOC/GPI/ENO1/PFKP/TPI1/GAPDH/GCK/IGFBP3/HK1/GALK1/PDK3/ALDOB/ENO3/GAPDHS/GAA/PKLR/RORA/PGM2/BRS3/TKTL1/PGAM2/SLC37A4/PPARGC1A/SLC25A1/FBP1/PCK1/FABP5/PPARD/TP53/PPP1CA/PTPN2/LEPR/DDB1/MAPK14/PMAIP1/ADIPOQ/SORBS1/SLC25A10/GPD2/LEP/DLAT/MDH2/IGFBP4/NPY1R/TFF3/DCXR/GALE/SORD/AKT1/ACTN3/AKT2/IGF1/PC/PFKM/APOD/OAS1/BPGM/IRS1/INPPL1/SRC/GHRL/PIK3CA/DYRK2/ATF4/SERP1/GSK3B/GOT1/G6PD/GOT2/FUCA1/SELENOS/PDK4/GNMT/RBP4/GCKR/TKFC/PMM1/G6PC/ARPP19/MLYCD/ALDH1A1/GPD1/MDH1/PDHB/PPARA/CPT1A/PDHA1/SLC25A11/COX11/SLC25A12/PKM/PFKFB1/PGAM1/PMM2/FBP2/MPI/AKR1A1/CHST1/SLC25A13/FUT8/GK2/ONECUT1/NFE2L1/ADIPOR1/RGN/MST1/ENPP1/GALM/PDHA2/PGK2/PHKG2/MTOR/POMC/SIRT1/IGF2/HKDC1/PDK2/PRKN/TFAP2B/FOXA2/INS/PDX1/FOXO1/G6PC2
GO	monosaccharide metabolic process	154/4318	287/18670	1.73E-29	1.61E-27	5.65E-28	ATF3/TNF/PHLDA2/PFKFB3/B4GALT1/FUT4/SIK1/IRS2/SLC2A3/PGK1/PDK1/PFKL/ALDOA/ENO2/PGM1/HK2/ALDOC/GPI/SLC2A1/ENO1/PFKP/TPI1/GAPDH/GCK/IGFBP3/HK1/GALK1/PDK3/ALDOB/ENO3/GAPDHS/GAA/PKLR/RORA/PGM2/BRS3/TKTL1/PGAM2/SLC37A4/PPARGC1A/SLC25A1/FBP1/PCK1/FABP5/PPARD/TP53/PPP1CA/PTPN2/LEPR/DDB1/MAPK14/PMAIP1/ADIPOQ/SORBS1/TALDO1/SLC25A10/GPD2/LEP/TKT/DLAT/MDH2/IGFBP4/NPY1R/TFF3/DCXR/GALE/SORD/AKT1/ACTN3/AKT2/IGF1/PC/PFKM/APOD/OAS1/BPGM/IRS1/INPPL1/SRC/GHRL/PIK3CA/DYRK2/ATF4/SERP1/GSK3B/GOT1/G6PD/GCLC/GOT2/FUCA1/SELENOS/CYB5A/GSTO1/PDK4/GNMT/RBP4/GCKR/TKFC/PMM1/G6PC/PGD/ARPP19/MLYCD/ALDH1A1/GPD1/MDH1/PDHB/PPARA/CPT1A/PDHA1/SLC25A11/COX11/CYB5R3/SLC25A12/PKM/PFKFB1/PGAM1/PMM2/FBP2/MPI/AKR1A1/CHST1/SLC25A13/FUT8/GK2/RPE/ONECUT1/PRKCE/NFE2L1/ADIPOR1/RGN/MST1/ENPP1/GALM/SLC23A2/SLC23A2/UGT2B17/PDHA2/PGK2/PHKG2/MTOR/POMC/SIRT1/IGF2/HKDC1/FGGY/PDK2/PRKN/TFAP2B/FOXA2/INS/PDX1/FOXO1/G6PC2
GO	glycosaminoglycan metabolic process	79/4318	160/18670	3.26E-13	3.95E-12	1.38E-12	IL1B/SDC4/NFKB1/CD44/B4GALT1/B4GALT5/EXT1/CHST2/BCAN/GPC3/NDST1/IDS/HS3ST1/GPC4/HEXA/BGN/PIM1/SDC3/CHST3/GCNT2/HAS1/SDC2/PDGFB/B3GALT6/DCN/NDST2/GPC1/NCAN/GUSB/TGFB1/HMMR/EGF/PDGFRB/HGF/LUM/ST3GAL6/ITIH5/OMD/ANGPT1/FOXC1/UGDH/AKT1/AGRN/FGF2/CLTC/IL15/VCAN/SPOCK2/ITIH1/SDC1/FUCA1/FMOD/HAS2/SLC35D1/ITIH4/B4GALT7/CHPF/B3GAT3/CHST12/HS2ST1/B3GAT1/CHPF2/CHST6/CHST1/EXT2/B4GALT2/IDUA/GLCE/HS6ST2/B3GNT3/CLN6/NDST3/B4GALT4/XYLT2/ABCC5/HYAL2/PGLYRP1/ST3GAL4/ITIH3
GO	polysaccharide metabolic process	55/4318	114/18670	3.60E-09	2.43E-08	8.49E-09	NFKB1/PHLDA2/B4GALT5/IL6ST/IRS2/GBE1/PGM1/GYS1/GCK/PYGM/EXT1/UGP2/NDST1/GAA/STBD1/PPP1R3C/PGM2/HAS1/PDGFB/PHKG1/NDST2/TGFB1/PPP1CA/LEPR/EGF/SORBS1/AKT1/AKT2/IGF1/AGL/CLTC/IRS1/CALM1/DYRK2/GSK3B/HAS2/SELENOS/GNMT/G6PC/PYGB/HS2ST1/CHST1/EXT2/PHKA2/GYS2/B3GNT3/PYGL/NDST3/PPP1R2/ENPP1/PHKG2/MTOR/POMC/IGF2/INS
GO	regulation of glucose metabolic process	54/4318	112/18670	5.13E-09	3.40E-08	1.19E-08	PHLDA2/SIK1/IRS2/PDK1/GCK/IGFBP3/PDK3/RORA/PPARGC1A/FBP1/TP53/PPP1CA/PTPN2/LEPR/DDB1/PMAIP1/ADIPOQ/SORBS1/LEP/IGFBP4/TFF3/AKT1/ACTN3/AKT2/IGF1/IRS1/SRC/DYRK2/GSK3B/SELENOS/PDK4/GNMT/GCKR/ARPP19/MLYCD/PPARA/COX11/SLC25A12/PFKFB1/NFE2L1/ADIPOR1/RGN/MST1/ENPP1/PHKG2/MTOR/POMC/SIRT1/IGF2/PDK2/PRKN/FOXA2/INS/FOXO1
GO	glycogen metabolic process	39/4318	74/18670	3.15E-08	1.84E-07	6.45E-08	PHLDA2/IL6ST/IRS2/GBE1/PGM1/GYS1/GCK/PYGM/UGP2/GAA/STBD1/PPP1R3C/PGM2/PHKG1/PPP1CA/LEPR/SORBS1/AKT1/AKT2/IGF1/AGL/IRS1/CALM1/DYRK2/GSK3B/SELENOS/GNMT/G6PC/PYGB/PHKA2/GYS2/PYGL/PPP1R2/ENPP1/PHKG2/MTOR/POMC/IGF2/INS
GO	cellular polysaccharide metabolic process	49/4318	103/18670	4.45E-08	2.53E-07	8.86E-08	PHLDA2/B4GALT5/IL6ST/IRS2/GBE1/PGM1/GYS1/GCK/PYGM/EXT1/UGP2/NDST1/GAA/STBD1/PPP1R3C/PGM2/HAS1/PHKG1/NDST2/PPP1CA/LEPR/SORBS1/AKT1/AKT2/IGF1/AGL/IRS1/CALM1/DYRK2/GSK3B/HAS2/SELENOS/GNMT/G6PC/PYGB/HS2ST1/EXT2/PHKA2/GYS2/B3GNT3/PYGL/NDST3/PPP1R2/ENPP1/PHKG2/MTOR/POMC/IGF2/INS
GO	cellular glucan metabolic process	39/4318	75/18670	5.10E-08	2.87E-07	1.01E-07	PHLDA2/IL6ST/IRS2/GBE1/PGM1/GYS1/GCK/PYGM/UGP2/GAA/STBD1/PPP1R3C/PGM2/PHKG1/PPP1CA/LEPR/SORBS1/AKT1/AKT2/IGF1/AGL/IRS1/CALM1/DYRK2/GSK3B/SELENOS/GNMT/G6PC/PYGB/PHKA2/GYS2/PYGL/PPP1R2/ENPP1/PHKG2/MTOR/POMC/IGF2/INS
GO	glucan metabolic process	39/4318	75/18670	5.10E-08	2.87E-07	1.01E-07	PHLDA2/IL6ST/IRS2/GBE1/PGM1/GYS1/GCK/PYGM/UGP2/GAA/STBD1/PPP1R3C/PGM2/PHKG1/PPP1CA/LEPR/SORBS1/AKT1/AKT2/IGF1/AGL/IRS1/CALM1/DYRK2/GSK3B/SELENOS/GNMT/G6PC/PYGB/PHKA2/GYS2/PYGL/PPP1R2/ENPP1/PHKG2/MTOR/POMC/IGF2/INS
GO	glycosyl compound metabolic process	57/4318	130/18670	1.33E-07	6.94E-07	2.43E-07	AK4/AMPD3/GUSB/DLG1/RHOA/NME1/NT5C3A/NME4/PNP/ADA/NME3/NT5C/APRT/IMPDH2/GUK1/DGUOK/TK2/HPRT1/CDA/UMPS/AK3/GMPR2/DTYMK/AMD1/UCK2/DPYD/UCK1/PEMT/GLA/TH/GNAI3/UPP1/GAMT/APOBEC3G/APOBEC3F/GMPS/TK2/RAN/DCK/PRPS1/CAD/PRPS2/ERH/FUCA1/NT5E/AKR1C3/UPB1/AHCY/GNMT/AKR1C2/OPA1/AKR1A1/PPAT/MPP1/CDADC1/CBR4/AKR1B10

GO	positive regulation of glucose metabolic process	23/4318	38/18670	8.45E-07	3.87E-06	1.35E-06	IRS2/GCK/PPARGC1A/PTPN2/DDB1/PMAIP1/SORBS1/AKT1/ACTN3/AKT2/IGF1/IRS1/SRC/DYRK2/ARPP19/PPARA/SLC25A12/RGN/PHKG2/SIRT1/IGF2/INS/FOXO1
GO	proteoglycan metabolic process	42/4318	94/18670	3.16E-06	1.30E-05	4.54E-06	BMP2/EXT1/BCAN/NDST1/IDS/HS3ST1/HEXA/BGN/CHST3/B3GALT6/DCN/NDST2/GPC1/NCAN/CTNNB1/PPARD/BMPR2/CANT1/TCF7L2/UGDH/CHST8/BMPR1B/IGF1/VCAN/SULF2/SPOCK2/COL11A1/SLC35D1/B4GALT7/CHPF/B3GAT3/CHST12/HS2ST1/B3GAT1/CHPF2/EXT2/IDUA/GLCE/HS6ST2/NDST3/XYLT2/COL2A1
GO	regulation of polysaccharide metabolic process	24/4318	43/18670	3.71E-06	1.50E-05	5.25E-06	NFKB1/PHLDA2/IRS2/GCK/PDGFB/TGFB1/PPP1CA/EGF/SORBS1/AKT1/AKT2/IGF1/CLTC/IRS1/DYRK2/GSK3B/HAS2/SELENOS/ENPP1/PHKG2/MTOR/POMC/IGF2/INS
GO	fructose 1,6-bisphosphate metabolic process	9/4318	10/18670	1.49E-05	5.28E-05	1.85E-05	PFKL/ALDOA/ALDOC/PFKP/ALDOB/FBP1/PFKM/FBP2/IFNG
GO	glucose 6-phosphate metabolic process	16/4318	25/18670	1.50E-05	5.28E-05	1.85E-05	HK2/GPI/GCK/HK1/PGM2/TALDO1/TKT/G6PD/G6PC/PGD/PGAM1/PGLS/RPE/NFE2L1/HKDC1/G6PC2
GO	regulation of glycogen metabolic process	18/4318	35/18670	0.0002477	0.000673437	0.000235889	PHLDA2/IRS2/GCK/PPP1CA/SORBS1/AKT1/AKT2/IGF1/IRS1/DYRK2/GSK3B/SELENOS/ENPP1/PHKG2/MTOR/POMC/IGF2/INS
GO	fructose metabolic process	10/4318	15/18670	0.00040412	0.001054491	0.000369363	PFKFB3/ALDOA/ALDOC/ALDOB/FBP1/SORD/TKFC/ALDH1A1/PFKFB1/FBP2
GO	galactose metabolic process	9/4318	13/18670	0.000531971	0.001350226	0.000472952	B4GALT1/PGM1/GALK1/PGM2/PPARGC1A/GALE/CHST1/GK2/GALM
GO	positive regulation of glycogen metabolic process	11/4318	18/18670	0.000610972	0.001531367	0.000536401	IRS2/GCK/SORBS1/AKT1/AKT2/IGF1/IRS1/DYRK2/PHKG2/IGF2/INS
GO	disaccharide metabolic process	8/4318	11/18670	0.000676294	0.001665823	0.000583497	B4GALT1/SLC2A1/GAA/FBP1/LALBA/FBP2/IDUA/BRAF
GO	glucosamine-containing compound metabolic process	11/4318	24/18670	0.011891653	0.021048203	0.007372678	CHST2/LARGE1/NAGK/CHST3/PGM3/GNPDA1/CHST6/CHST1/GNE/NANP/CHST4
GO	glycoprotein metabolic process	114/4318	419/18670	0.027484567	0.043647559	0.015288688	HBEGF/PTX3/PHLDA1/TNIP1/B4GALT1/FUT4/B4GALT5/BMP2/GFPT2/EXT1/BCAN/NDST1/LARGE1/IDS/HS3ST1/HEXA/BGN/ATP7A/CHST3/B4GALNT2/BCL2/GCNT2/B3GALT6/DCN/NDST2/GPC1/NCAN/MVD/FBXO6/CTNNB1/ARFGEF1/PPARD/NCSTN/BMPR2/CANT1/DAD1/HIF1A/PSEN1/TCF7L2/LFNG/ST3GAL6/LEP/VEGFB/MUC1/AMFR/UGDH/CHST8/ST6GALNAC2/BMPR1B/NGLY1/UBE2J1/PGM3/LMAN1/IGF1/IL15/ST3GAL5/ST8SIA4/VCAN/SULF2/SPOCK2/MT3/MMP12/SERP1/EDEM1/PLCB1/RAB1A/RPN1/SDF2L1/COL11A1/PLOD3/CCR7/SLC35D1/PMM1/NPC1/ENTPD5/MAN1A1/MARCHF6/ACOT8/OSTC/PMM2/B4GALT7/CHPF/B3GAT3/CHST12/HS2ST1/MPI/B3GAT1/CHPF2/ALG1/GFPT1/EXT2/B4GALT2/FUT8/IDUA/GLCE/HS6ST2/SRD5A3/B3GNT3/GAL3ST1/NDST3/B4GALT4/XYLT2/CHST4/AGO2/MGAT1/COL2A1/GCNT1/TRAK2/GATA1/ST3GAL4/CCL19/GALNT1/GALNT3/ST6GAL1
GO	positive regulation of glycoprotein metabolic process	10/4318	24/18670	0.033459661	0.052512851	0.018393987	CTNNB1/ARFGEF1/NCSTN/TCF7L2/IGF1/IL15/PLCB1/RAB1A/CCR7/CCL19
GO	pentose metabolic process	7/4318	15/18670	0.038431909	0.05859725	0.020525205	TALDO1/TKT/DCXR/G6PD/PGD/RPE/FGGY
GO	regulation of glycoprotein metabolic process	17/4318	49/18670	0.044193094	0.066999402	0.023468276	HBEGF/PTX3/BCL2/CTNNB1/ARFGEF1/NCSTN/TCF7L2/IGF1/IL15/MT3/PLCB1/RAB1A/CCR7/ACOT8/AGO2/GATA1/CCL19
GO	oligosaccharide metabolic process	14/4318	48/18670	0.202621339	0.25254881	0.088461764	B4GALT1/SLC2A1/GAA/FBP1/LALBA/ST3GAL6/GLA/ST8SIA4/FBP2/FUT8/IDUA/GM2A/ST3GAL4/BRAF
GO	glycosphingolipid metabolic process	17/4318	66/18670	0.35063512	0.410627765	0.143833013	B4GALT5/LARGE1/HEXA/BAX/ESYT1/UGCG/GLA/GALC/ST3GAL5/ST8SIA4/PRKCD/ITGB8/CLN6/GAL3ST1/GM2A/KIT/STS

Supplementary Table 2

gene_id (DE-GRGs)

CD44	VCAN
GALNT3	NCAN
UGT2B17	FBP2
ALDOC	BCAN
CCL19	AKR1C2
FOXA2	STBD1
MT3	AHCY
TFAP2B	TH
PPARGC1A	PRKCE
TNF	RBP4
MMP12	PLCB1
CCR7	SPOCK2
PRKN	COL2A1
INS	UPP1
APOD	PYGM
GCKR	

The top 10 significant items (GO-BP, CC, MF and KEGG) were shown here

ONTOLOGY	ID	Description	GeneRatio	BgRatio	pvalue	p.adjust	qvalue	geneID
BP	GO:0005996	monosaccharide metabolic process	13/31	292/18670	4.11456E-16	8.6735E-13	4.13189E-13	ALDOC/APOD/GCKR/FOXA2/INS/PRKN/PRKCE/RBP4/TFAP2B/TNF/UGT2B17/FBP2/PPARGC1A
BP	GO:0006006	glucose metabolic process	11/31	209/18670	1.84886E-14	1.9487E-11	9.28323E-12	ALDOC/APOD/GCKR/FOXA2/INS/PRKN/RBP4/TFAP2B/TNF/FBP2/PPARGC1A
BP	GO:0019318	hexose metabolic process	11/31	249/18670	1.27408E-13	8.95255E-11	4.26482E-11	ALDOC/APOD/GCKR/FOXA2/INS/PRKN/RBP4/TFAP2B/TNF/FBP2/PPARGC1A
BP	GO:0009100	glycoprotein metabolic process	11/31	419/18670	3.61071E-11	1.90284E-08	9.06478E-09	CCR7/COL2A1/VCAN/NCAN/GALNT3/MMP12/MT3/CCL19/SPOCK2/PLCB1/BCAN
BP	GO:0044262	cellular carbohydrate metabolic process	9/31	286/18670	6.14793E-10	2.59197E-07	1.23476E-07	GCKR/FOXA2/INS/PRKN/PRKCE/PYGM/FBP2/STBD1/PPARGC1A
BP	GO:0050708	regulation of protein secretion	10/31	472/18670	2.67263E-09	9.38984E-07	4.47314E-07	CCR7/FOXA2/INS/MMP12/PRKN/PRKCE/RBP4/CCL19/TFAP2B/TNF
BP	GO:0042593	glucose homeostasis	8/31	241/18670	4.19075E-09	1.08769E-06	5.18157E-07	GCKR/FOXA2/INS/PRKCE/RBP4/TFAP2B/TH/PPARGC1A
BP	GO:0033500	carbohydrate homeostasis	8/31	242/18670	4.32924E-09	1.08769E-06	5.18157E-07	GCKR/FOXA2/INS/PRKCE/RBP4/TFAP2B/TH/PPARGC1A
BP	GO:0002791	regulation of peptide secretion	10/31	500/18670	4.64386E-09	1.08769E-06	5.18157E-07	CCR7/FOXA2/INS/MMP12/PRKN/PRKCE/RBP4/CCL19/TFAP2B/TNF
BP	GO:0010677	negative regulation of cellular carbohydrate metabolic process	5/31	42/18670	7.32834E-09	1.54481E-06	7.3592E-07	GCKR/FOXA2/INS/PRKN/PPARGC1A
MF	GO:0031406	carboxylic acid binding	6/31	193/17697	9.13337E-07	7.35879E-05	4.78435E-05	CD44/VCAN/NCAN/AKR1C2/TH/BCAN
MF	GO:0043177	organic acid binding	6/31	205/17697	1.29861E-06	7.35879E-05	4.78435E-05	CD44/VCAN/NCAN/AKR1C2/TH/BCAN
MF	GO:0030246	carbohydrate binding	6/31	271/17697	6.51188E-06	0.000276755	0.000179934	VCAN/NCAN/GALNT3/GCKR/STBD1/BCAN
MF	GO:0005539	glycosaminoglycan binding	5/31	229/17697	4.48415E-05	0.001524611	0.000991233	CD44/VCAN/NCAN/SPOCK2/BCAN
MF	GO:0005540	hyaluronic acid binding	4/31	21/17697	4.51475E-08	7.67507E-06	4.98998E-06	CD44/VCAN/NCAN/BCAN
MF	GO:0016757	transferase activity, transferring glycosyl groups	4/31	280/17697	0.001378343	0.024356289	0.015835358	GALNT3/PYGM/UGT2B17/UPP1
MF	GO:0004857	enzyme inhibitor activity	4/31	375/17697	0.00397143	0.045009535	0.029263165	GCKR/MT3/TFAP2B/SPOCK2
MF	GO:0005518	collagen binding	3/31	67/17697	0.000216105	0.006122976	0.003980882	CD44/MMP12/SPOCK2
MF	GO:0043178	alcohol binding	3/31	85/17697	0.000436164	0.010592545	0.006886794	APOD/PRKCE/RBP4
MF	GO:0002020	protease binding	3/31	128/17697	0.001432723	0.024356289	0.015835358	INS/PRKN/TNF
CC	GO:0005796	Golgi lumen	4/31	102/19717	1.90766E-05	0.002003047	0.00150605	VCAN/NCAN/INS/BCAN
CC	GO:0043202	lysosomal lumen	3/31	95/19717	0.000441656	0.02318695	0.017433797	VCAN/NCAN/BCAN
CC	GO:0005775	vacuolar lumen	3/31	172/19717	0.002450375	0.085763111	0.064483542	VCAN/NCAN/BCAN
KEGG	hsa04931	Insulin resistance	5/21	108/8076	6.68739E-06	0.001103419	0.000893998	INS/PRKCE/PYGM/TNF/PPARGC1A
KEGG	hsa04922	Glucagon signaling pathway	4/21	107/8076	0.000146542	0.011775086	0.009540261	PYGM/FBP2/PPARGC1A/PLCB1
KEGG	hsa04930	Type II diabetes mellitus	3/21	46/8076	0.000214092	0.011775086	0.009540261	INS/PRKCE/TNF
KEGG	hsa04910	Insulin signaling pathway	4/21	137/8076	0.000379053	0.015635929	0.012668345	INS/PYGM/FBP2/PPARGC1A
KEGG	hsa04950	Maturity onset diabetes of the young	2/21	26/8076	0.002015806	0.048125166	0.038991363	FOXA2/INS
KEGG	hsa04061	Viral protein interaction with cytokine and cytokine receptor	3/21	100/8076	0.002083683	0.048125166	0.038991363	CCR7/CCL19/TNF
KEGG	hsa04933	AGE-RAGE signaling pathway in diabetic complications	3/21	100/8076	0.002083683	0.048125166	0.038991363	PRKCE/TNF/PLCB1
KEGG	hsa00030	Pentose phosphate pathway	2/21	30/8076	0.002681233	0.048125166	0.038991363	ALDOC/FBP2
KEGG	hsa00051	Fructose and mannose metabolism	2/21	33/8076	0.003239215	0.048125166	0.038991363	ALDOC/FBP2
KEGG	hsa05131	Shigellosis	4/21	246/8076	0.003340765	0.048125166	0.038991363	CD44/PRKCE/TNF/PLCB1

Supplementary Table 3

Primer name	Sequence
ALKBH5-forward	CCAGCTATGCTTCAGATCGCCT
ALKBH5-reverse	GGTTCTCTTCCTTGTCCATCTCC
AHCY-forward	ATCCTCAAGGTGCCTGCCATCA
AHCY-reverse	CGGCAATCATCACATCTGTGGC
NCAN-forward	CCAATGAGGTGGATATGCCTGG
NCAN-reverse	TCGGCTTTCTCTAGCTCCAGGA
FBP2-forward	CTCCATCGGAACCATCTTTGCC
FBP2-reverse	GTTGCACTACCGTACAGCGCAT
GALNT3-forward	GTTGCTAGGAGCAACAGTCGCA
GALNT3-reverse	CTGGACTTACGACAGCCGTGTA
AKR1C2-forward	CAGTGGATCTCTGTGCCACATG
AKR1C2-reverse	CTGGTTGCAGACAGGCTTGTAC
GAPDH-forward	TGACTTCAACAGCGACACCCA
GAPDH-reverse	CACCCTGTTGCTGTAGCCAAA
**Target oligo**	**Sequence**
shALKBH5-1#-F	CCGGCCACCCAGCTATGCTTCAGATCTCGAGATCTGAAGCATAGCTGGGTGGTTTTTG
shALKBH5-1#-R	AATTCAAAAACCACCCAGCTATGCTTCAGATCTCGAGATCTGAAGCATAGCTGGGTGG
shALKBH5-2#-F	CCGGGAAAGGCTGTTGGCATCAATACTCGAGTATTGATGCCAACAGCCTTTCTTTTTG
shALKBH5-2#-R	AATTCAAAAAGAAAGGCTGTTGGCATCAATACTCGAGTATTGATGCCAACAGCCTTTC
