# Supplementary material for: Identification and Characterization of a Glucometabolic Prognostic Gene Signature in Neuroblastoma based on N6-methyladenosine Eraser ALKBH5
Source: J Cancer. 2022 Mar 28;13(7):2105–25. doi: 10.7150/jca.69408 (PMC9066222; doi:10.7150/jca.69408)
Supplement: Supplementary file 2 — Supplementary figures. [file jcav13p2105s2.pdf]

Supplemental Figure 1

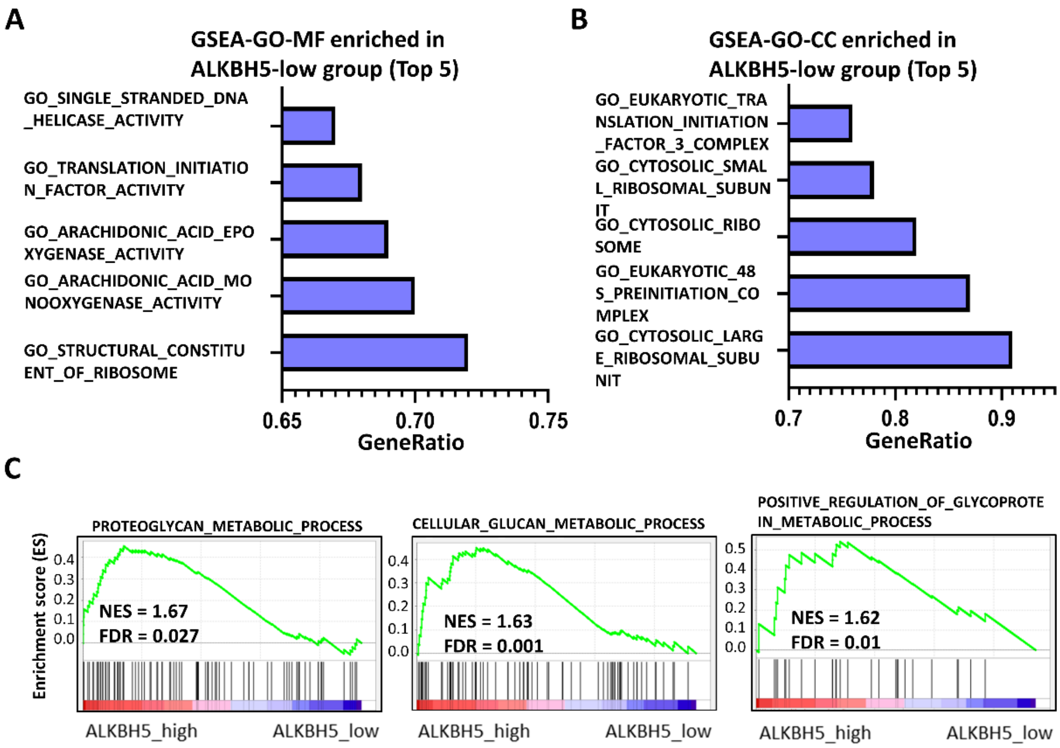

Figure S1

Identification and Landscape of ALKBH5

(**A, B**) Top 5 GO-MF (A) and CC (B) gene sets enriched in the ALKBH5-low group obtained by GSEA. (**C**) Three additional representative glucometabolic gene sets enriched in the ALKBH5-high group. NES: normalized enrichment score. FDR: false discovery rate.

Supplemental Figure 2

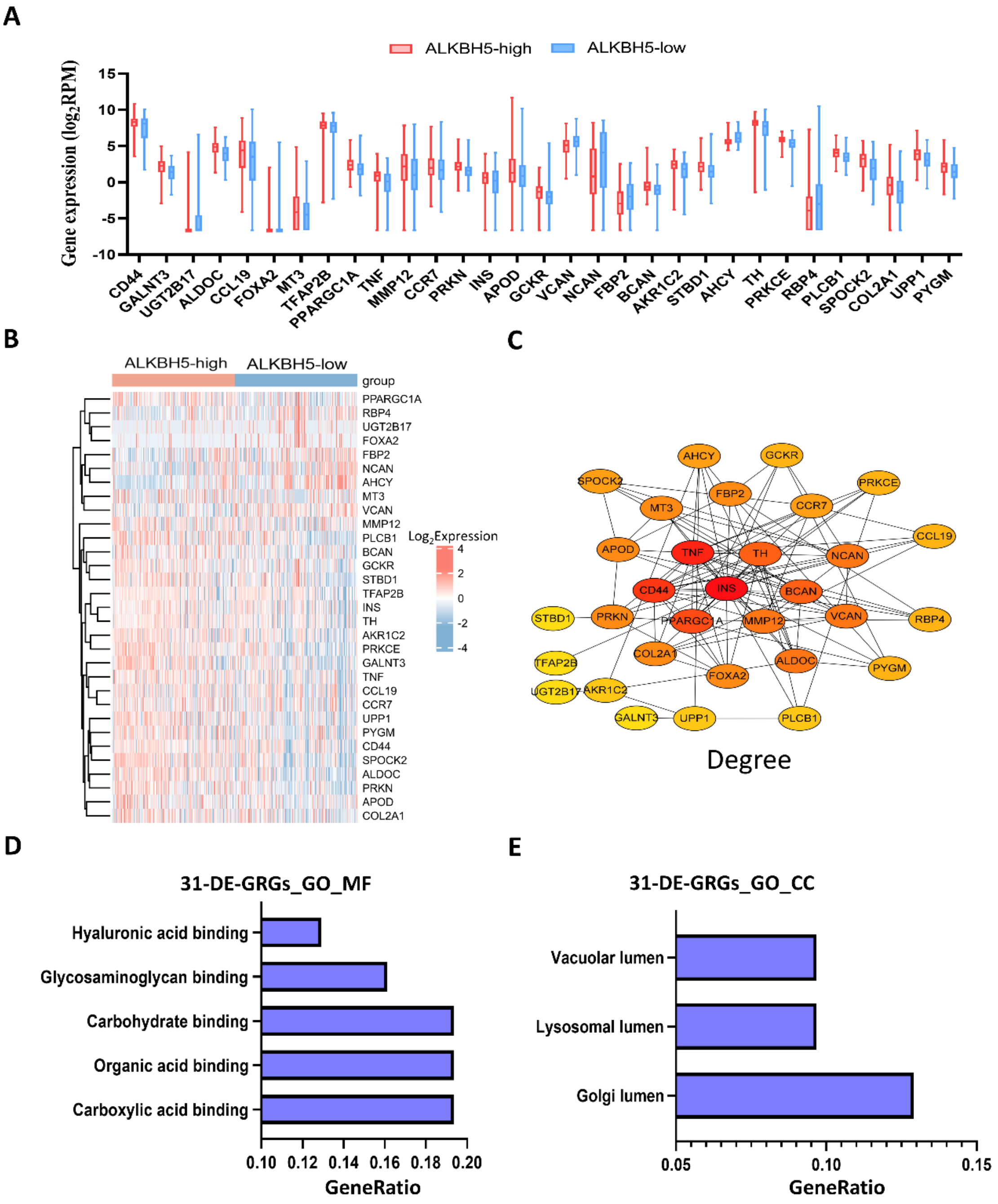

Figure S2

Identification and Functional Enrichment Analyses of DE-GRGs

(**A, B**) Gene expression profile of 31 DE-GRGs in the boxplot (A) and heatmap (B). All difference reaches statistical significance. (**C**) Thirty-one DE-GRGs in the PPI network calculated by Degree. (**D, E**) Top 5 and 3 gene sets functionally enriched by GO-MF (D) and CC (E) annotations.

Supplemental Figure 3

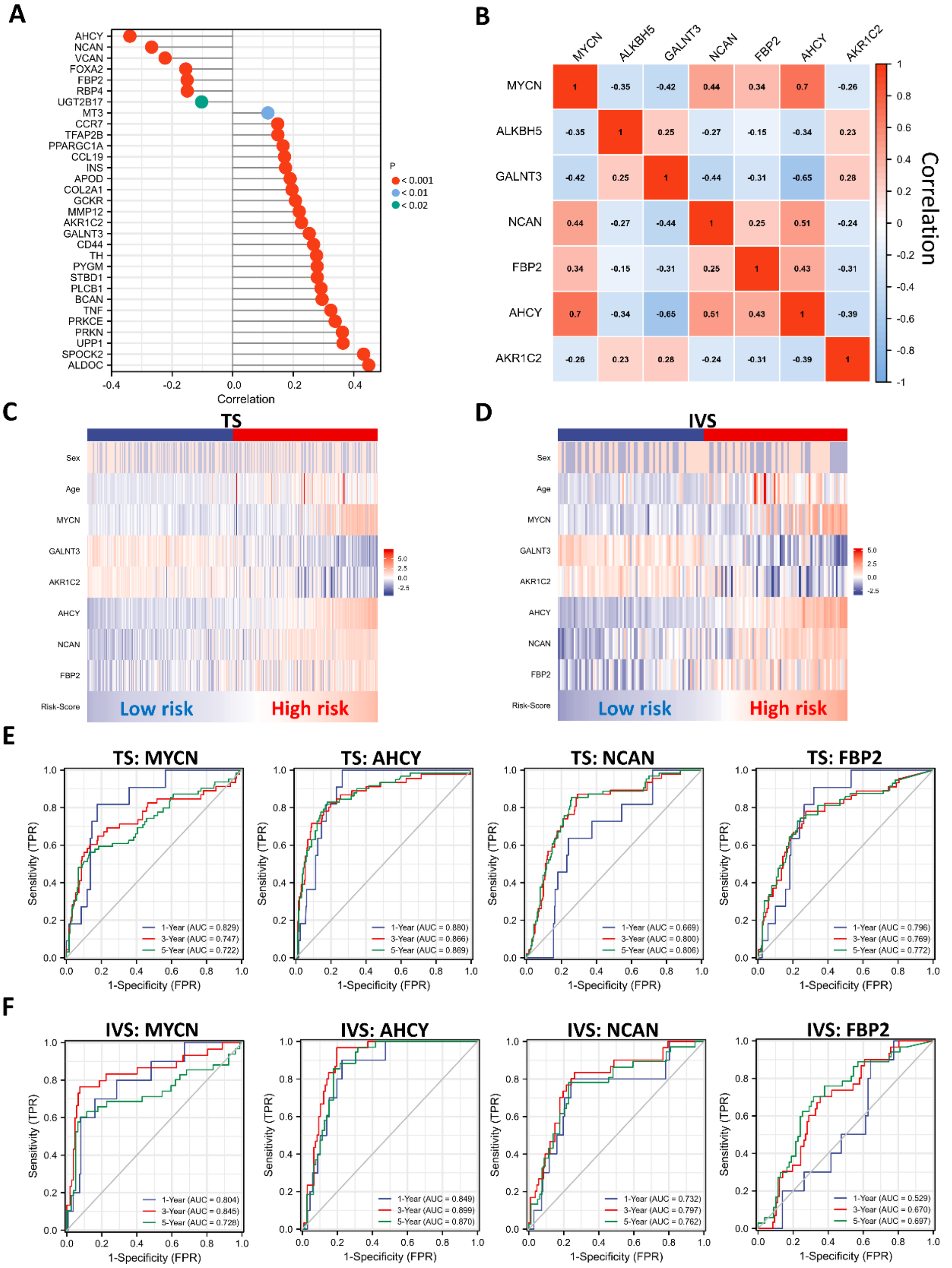

Figure S3

Five-gene Prognostic Signature Based on 31 DE-GRGs

(**A**) Correlation analysis between ALKBH5 and 31 DE-GRGs in GSE62564. (**B**) Correlation analysis between MYCN/ALKBH5 and genes in the model in GSE62564. (**C, D**) Heatmap showing the profile of clinical characteristics and the 5-gene model in TS (C) and IVS (D). (**E, F**) Time-dependent ROC curve in TS (E) and IVS (F) for independent risk factors including MYCN, AHCY, NCAN and FBP2. TS: training set. IVS: internal validation set. AUC: area under curve.

Supplemental Figure 4

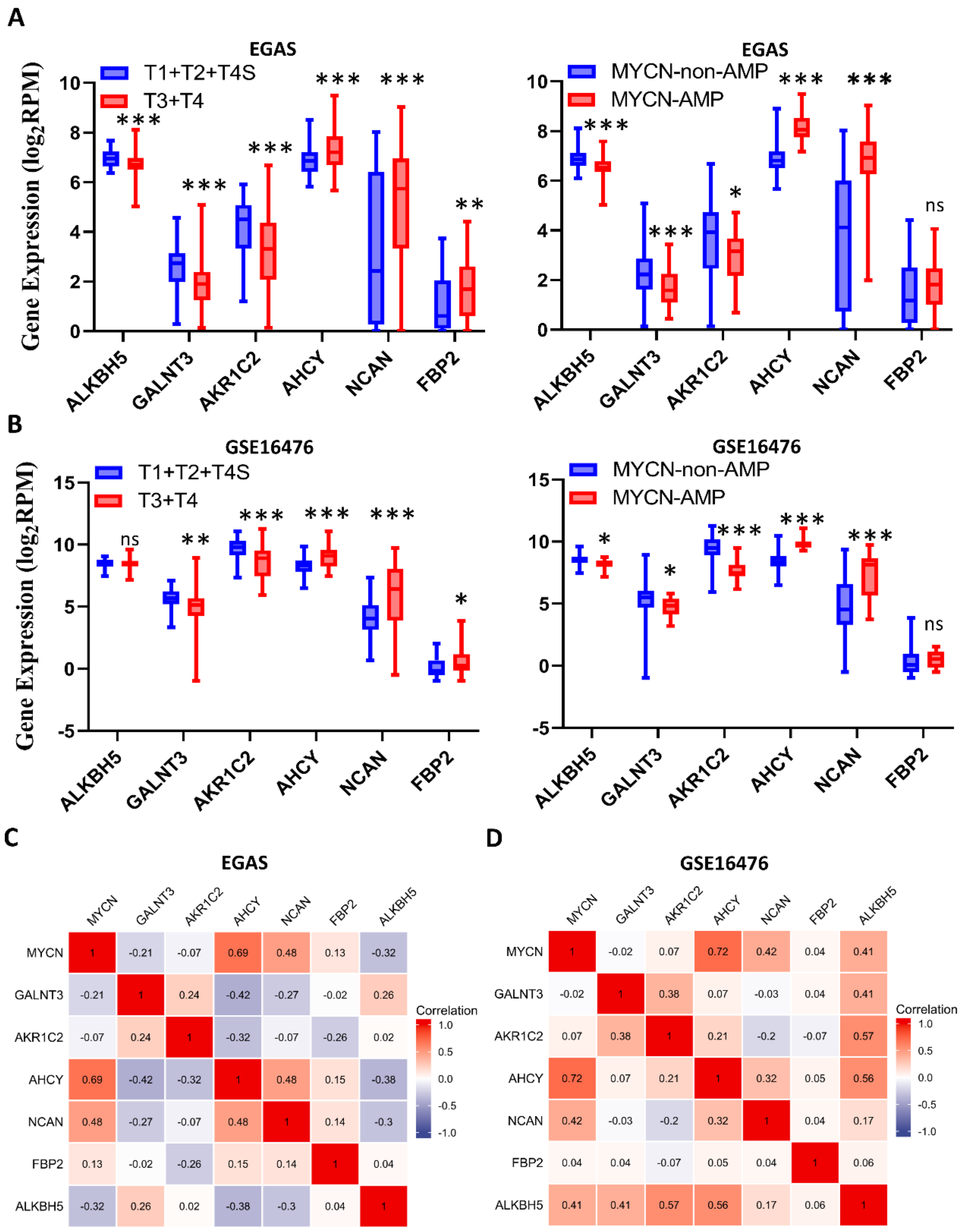

Figure S4

External Validation of the Gene Signature with Public Datasets

(**A, B**) Gene expression profile of ALKBH5 and 5 genes in the model based on INSS and MYCN amplification status from EGAS (A) and GSE16476 (B). (**C, D**) Matrix showing gene-gene correlation value among MYCN, ALKBH5 and other 5 genes in the model in EGAS (C) and GSE16476 (D). NS: not significant. P<0.05 was shown as *, P<0.01 as ** and P<0.001 as ***.

Supplemental Figure 5

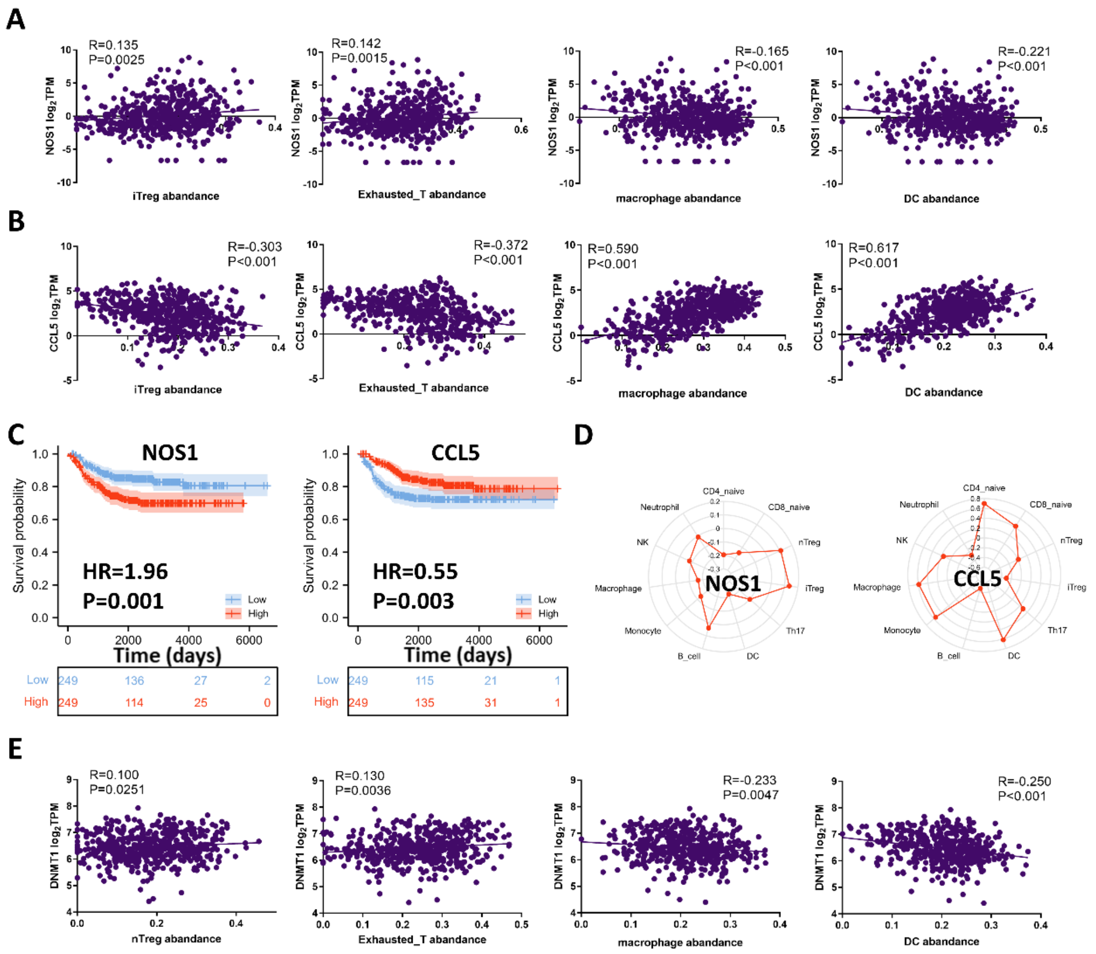

Figure S5

Immune Landscape of Neuroblastoma Patients and Potential Targets

(**A, B**) Pearson correlation analysis between NOS1 (A)/CCL5 (B) and iTregs/Exhausted T cells/macrophages/DCs. (**C**) Kaplan-Meier curve of GSE62564 between the NOS1-high and NOS1-low groups (left panel), the CCL5-high and CCL5-low groups (right panel). (**D**) Radar map showing relationship between immune cells and NOS1 (left panel)/CCL5 (right panel). (**E**) Pearson correlation analysis between DNMT1 and nTregs/Exhausted T cells/macrophages/DCs. nTregs: natural T regulatory cells. iTregs: inducible T regulatory cells. DCs: dendritic cells.
